# Human Immunodeficiency Virus Drug Resistance: 2018 Recommendations of the International Antiviral Society–USA Panel

**DOI:** 10.1093/cid/ciy463

**Published:** 2018-07-20

**Authors:** Huldrych F Günthard, Vincent Calvez, Roger Paredes, Deenan Pillay, Robert W Shafer, Annemarie M Wensing, Donna M Jacobsen, Douglas D Richman

**Affiliations:** 1 University Hospital Zürich and Institute of Medical Virology, University of Zurich, Switzerland; 2 Pierre et Marie Curie University and Pitié-Salpêtrieŕe Hospital, Paris, France; 3 Infectious Diseases Service and IrsiCaixa AIDS Research Institute, Hospital Universitari Germans Trias i Pujol, Badalona, Spain; 4 Africa Health Research Institute, KwaZulu Natal, South Africa; 5 University College London, United Kingdom; 6 Stanford University Medical School, California; 7 University Medical Center Utrecht, The Netherlands; 8 International Antiviral Society–USA, San Francisco; 9 Veterans Affairs San Diego Healthcare System and University of California San Diego

**Keywords:** HIV, antiretroviral therapy, drug resistance, therapeutic failure, resource-rich, low and middle income countries, HIV-1 subtype, genotypic drug resistance, sanger sequencing, next generation sequencing

## Abstract

**Background:**

Contemporary antiretroviral therapies (ART) and management strategies have diminished both human immunodeficiency virus (HIV) treatment failure and the acquired resistance to drugs in resource-rich regions, but transmission of drug-resistant viruses has not similarly decreased. In low- and middle-income regions, ART roll-out has improved outcomes, but has resulted in increasing acquired and transmitted resistances. Our objective was to review resistance to ART drugs and methods to detect it, and to provide updated recommendations for testing and monitoring for drug resistance in HIV-infected individuals.

**Methods:**

A volunteer panel of experts appointed by the International Antiviral (formerly AIDS) Society–USA reviewed relevant peer-reviewed data that were published or presented at scientific conferences. Recommendations were rated according to the strength of the recommendation and quality of the evidence, and reached by full panel consensus.

**Results:**

Resistance testing remains a cornerstone of ART. It is recommended in newly-diagnosed individuals and in patients in whom ART has failed. Testing for transmitted integrase strand-transfer inhibitor resistance is currently not recommended, but this may change as more resistance emerges with widespread use. Sanger-based and next-generation sequencing approaches are each suited for genotypic testing. Testing for minority variants harboring drug resistance may only be considered if treatments depend on a first-generation nonnucleoside analogue reverse transcriptase inhibitor. Different HIV-1 subtypes do not need special considerations regarding resistance testing.

**Conclusions:**

Testing for HIV drug resistance in drug-naive individuals and in patients in whom antiretroviral drugs are failing, and the appreciation of the role of testing, are crucial to the prevention and management of failure of ART.

## Teaser

Testing for human immunodeficiency virus resistance in drug-naive individuals and in patients in whom antiretroviral treatment (ART) is failing, and the appreciation of the role of testing, are crucial to the prevention and management of failure of ART.

Human immunodeficiency virus (HIV) drug resistance is an important contributor to failure of antiretroviral therapies (ART). In resource-rich regions, acquired HIV drug resistance as a result of treatment failure has diminished with the availability of better drugs and better monitoring for treatment failure and drug resistant viruses [1]. Transmission of drug-resistant virus, however, persists at prevalences of around 10% in resource-rich countries. The rollout of ART in low- and middle-income countries (LMIC) reduced morbidity and mortality [2], but over the last few years prevalences of 10% or higher of transmitted drug resistance (TDR) have been reached in some countries [3–7].

This report [8] examines recent information regarding HIV drug resistance in resource-rich and LMIC, and provides updated recommendations [8, 9]. Drug-resistant mutations (DRMs) that impact treatment responses are updated regularly [9]. Implementation of next-generation sequencing methods is increasing and has changed approaches to drug resistance testing. These new approaches, and the insights they provide (eg, minority drug-resistant variants), are also addressed. Table 1 provides a summary of definitions of terms relevant for HIV drug resistance.

**Table 1.** Definitions of Terms Used in the Field of HIV Drug Resistance Testing

Term	Definition
Drug-resistance mutations	Specific mutations in the HIV-genome that are associated with a reduced in vitro and in vivo activity of antiretroviral drugs. DRMs are drug-class specific and generally emerge in the gene, which is targeted by the specific antiretroviral drug.
Acquired drug resistance	DRMs that are selected during antiretroviral therapy, which can happen when viral replication is not fully suppressed in the presence of drug.
Transmitted drug resistance	DRMs that are transmitted by (1) patients with acquired drug resistance or, (2) drug-naive patients who were infected with a resistant HIV strain.
Pretreatment drug resistance	All DRMs in patients before starting a treatment. TDRs, as well as DRMs from exposure to earlier ART, are included. Previous ART exposure, in particular in resource-limited settings, is often not disclosed; thus, TDR cannot reliably be determined in some settings.
HIV drug resistance by genotyping	Genotyping identifies DRMs in the HIV genome (that are associated with reduced activity of antiretroviral drugs).
HIV drug resistance by phenotyping	Phenotyping for drug resistance assesses the ability of a virus to grow in different concentrations of ARVs. Such resistance characterizes the reduced activity of ARVs to inhibit growth of a virus in vitro.
Viral tropism	Viral tropism is defined as the ability of HIV to selectively attach to a particular coreceptor on the surface of a host CD4 T-cell. The virus can attach either to the CCR5 coreceptor (R5-tropic), to the CXCR4 coreceptor (X4-tropic), or both (dual-tropic).
HIV-1 *pol*	The HIV-1 polymerase is a gene that encodes for viral structural proteins, namely the viral enzymes protease, reverse transcriptase and RNase H, and integrase.
HIV-1 *env*	The HIV-1 envelope gene encodes for the glycoprotein 160 protein, which is cleaved into gp120 and gp41. Those glycoprotein components mediate binding and entry into the target cell.
HIV-1 *gag*	The HIV-1 gag, or group-specific antigen, encodes for a number of structural proteins relevant for the viral structure (matrix protein, p17; capsid protein, p24; spacer peptide 1, p2; nucleocapsid protein, p7; spacer peptide 2, p1) and p6 protein.
Sanger sequencing	It has been used as the sequencing method of choice for commercially-available genotypic resistance testing. It is a DNA sequencing method used following reverse transcription of the viral ribonucleic acid genome, and is based on the selective incorporation of chain-terminating dideoxynucleotides by DNA polymerase during in vitro DNA replication. It requires a single-stranded DNA template, a DNA primer, a DNA polymerase, normal deoxynucleosidetriphosphates, and modified di-deoxynucleotidetriphosphates, the latter of which terminate DNA strand elongation. Today, mostly *Dye-terminator sequencing* is used. Each of the 4 dideoxynucleotide chain terminators are labelled with fluorescent dyes, each of which emits light at different wavelengths.
Next-generation sequencing	These newer technologies most likely will replace Sanger sequencing–based resistance testing within the next few years in research and commercial labs. Next-generation sequencing refers to high-throughput DNA sequencing technologies. Millions of DNA strands can be sequenced in parallel, yielding substantially more throughput and minimizing the need for the fragment-cloning methods that are often used in Sanger sequencing of genomes.
Point mutation assays	These assays are designed to detect predefined, known DRMs. They are primarily based on hybridization techniques, are relatively inexpensive, and only need simple laboratory equipment.
Home brew assays	These assays (eg, genotypic resistance testing assays) are developed by research groups or diagnostic labs and are not commercially available.

Abbreviations: ART, antiretroviral therapy; ARV; antiretroviral drugs; DRM, drug resistance mutation; gp, glycoprotein; HIV, human immunodeficiency virus; TDR, transmitted drug resistance.

## EPIDEMIOLOGY, ORIGIN, AND EFFECT OF TRANSMITTED DRUG RESISTANCE

### Prevalence of Transmitted Drug Resistance in Resource-rich Countries

Recommendations are provided in Appendix Box 1. TDR has been observed in virtually all countries where drug-resistance testing has been performed [3]. Frequencies vary substantially over time and by country. The prevalence of TDR is highest for nucleoside analogue reverse transcriptase inhibitors (nRTIs) and nonnucleoside analogue reverse transcriptase inhibitors (NNRTIs); is lower for protease inhibitors (PIs); and, so far, is rare for integrase strand transfer inhibitors (InSTIs). Frequencies may vary by location and time of testing. Table 2 summarizes the prevalence of TDR in some recent studies in seroconverter cohorts or in drug-naive individuals.

**Table 2.** Summary of Selected Prevalence Studies of TDR Mutations in Resource-rich Settings

Study Name and Citation	Country/Region	Sample Size	Drug-naive Population (Years Studied)	Prevalence of TDR Against Any Respectively Specific Drug Classes [No. (%)]				
				Any	NNRTI	nRTI	PI	InSTI
SPREAD Program [10]	Europe	4140	Chronically infected^a,b^ (2008–2010)	344 (8.3)	120 (2.9)%	186 (4.5)	83 (2)	NR
Robert Koch Institute [11]	Germany	809	Recently infected (2013–2014)	87 (10.8)%	21 (2.6)	37 (4.6)	24 (3)	NR
UK-CHIC [12]	United Kingdom	3 527 101 for InSTI testing	Chronically^a^ infected (2013)	233 (6.6)	116 (3.3)	124 (3.5)	60 (1.7)	0 (0)
ANRS PRIMO study [13]	France	1318	Recently infected (2007–2012)	154 (11.7)	51 (3.9)	69 (5.2)	33 (2.5)	NR
START Trial [14]	Europe/United States/ Australia	1869^c,d^	Chronically^a^ infected (2009–2013)	188 (10.1)	85 (4.5)	75 (4)	52 (2.8)	NR
CASCADE [15]	Europe (95%), Canada (1%), Australia 1%), sub-Saharan Africa (3%)	4717	Recently infected (1996–2012)	515 (11)	185 (3.9)	280 (5.9)	144 (3.1)	NR
SHCS [16]	Switzerland	1316	Chronically^a^ infected (2008–2014)	NR	NR	NR	NR	0 (0)
Stekler et al., 2015 [17]	United States (Seattle)	82	Chronically^a^ infected (2007–2012)	NR	NR	NR	NR	0 (0)

Abbreviations: ANRS PRIMO, French National Agency for Research on AIDS; CASCADE, concerted action on Seroconversion to AIDS and death in Europe; InSTI, integrase strand transfer inhibitor; NNRTI, non-nucleoside reverse transcriptase inhibitor; NR, not reported; nRTI, nucleoside reverse transcriptase inhibitor; PI, protease inhibitor; SHCS, the Swiss HIV cohort study; SPREAD, strategy to control SPREAD of HIV drug resistance; START, strategic timing of antiretroviral treatment; TDR, transmitted drug resistance; UK-CHIC, The UK collaborative HIV cohort.

^a^Duration of infection was not known.

^b^13% of patients were recently infected.

^c^Pretreatment resistance tests were available for 41.5% of all patients enrolled. Resistance tests were from Europe (69%), the United States (21%), Australia (5%), Asia (4%), and South America (1%).

^d^Enrollment for START was conducted on all continents.

The prevalence of TDR ranged from 6.6% to 11% and time trends seem to be rather stable, except in the United Kingdom, where TDR prevalence peaked in 2002 at 14% and dropped to 8% to 9% in 2009 [18, 19] and to 6.6% in 2013 [12]. In the Swiss HIV Cohort Study, the yearly TDR prevalence in recently infected patients fluctuated between 2.2% and 15.5% from 2000 to 2013, attributed in part to the introduction of new drugs (eg, boosted PIs in 2000 and InSTIs in 2008), after which substantial transient declines occurred [20]. Data on the transmission of InSTI resistances are scant. The larger studies, to date from the Swiss HIV Cohort Study and the United Kingdom, did not find any transmitted major InSTI DRMs since the class was introduced in 2007, despite the fact that thousands of patients are being treated with these drugs [12, 16, 17]. Anecdotal cases, however, have been reported [21, 22]. Thus, as seen for all drugs used at high frequency, InSTI TDR likely will increase over time.

### Prevalence of Transmitted or Pretreatment Drug Resistance in Low- and Middle-income Countries

With expanding use of ART in LMIC, acquired and transmitted drug resistance have increased [3–7]. It is increasingly recognized that a considerable proportion of detected DRMs in individuals in LMIC, previously assumed to be ART-naive, resulted from undisclosed exposure to earlier ART, rather than TDR [23]. Thus in LMIC, pretreatment drug resistance (PDR) data are more feasible to collect than TDR data, and remain important for treatment decisions.

Table 3 summarizes selected recent studies of PDR in LMIC. Surveillance conducted by the World Health Organization (WHO) between 2015 and 2016 in 11 countries from Africa, middle/south America, and Asia found that Uganda, Namibia, Zimbabwe, Nicaragua, Guatemala, and Argentina were above the critically-defined NNRTI TDR/PDR threshold of 10% [23]. A meta-analysis of 56044 adults with HIV infection from 64 LMIC estimated annual increases of the odds of PDR for NNRTIs of 23% (95% confidence interval [CI], 16–29) in southern Africa, 17% (95% CI, 5–30) in eastern Africa, 17% (95% CI, 6–29) in western and central Africa, 11% (95% CI, 5–18) in Latin America and the Caribbean, and 11% (95% CI, 2–20) in Asia [5]. Estimated absolute increases in prevalence of PDR between 2015 and 2016 ranged from 0.3% in Asia to 1.8% in southern Africa.

**Table 3.** Summary of Selected Prevalence Studies of Pretreatment Drug Resistance in Resource-limited Settings

Study Name and Citation	Country/Region	Sample Size	Drug-naive Population (Years Studied)	Prevalence of PDR Against Any Respectively Specific Drug Classes^a^				
				Any	NNRTI	nRTI	PI	InSTI
WHO [23]	Cameroon	321	Chronic (2015–2016)	24 (8.3)	23 (8.1)	5 (2.4)	1 (0.2)	NR
WHO [23]	Namibia	383	Chronic (2015–2016)	56 (14.6)	52 (13.8)	6 (1.6)	2 (0.5)	NR
WHO [23]	Uganda	342	Chronic (2016)	48 (17.4)	43 (15.4)	11 (5.1)	2 (1.0)	NR
WHO [23]	Zimbabwe	353	Chronic (2015–2016)	34 (10.9)	34 (10.9)	3 (0.8)	0 (0)	NR
WHO [23]	Guatemala	241	Chronic (2016)	34 (15.1)	29 (13.2)	9 (3.2)	2 (0.6)	NR
WHO [23]	Mexico	260	Chronic (2015–2016)	34 (13.5)	22 (9.2)	14 (5.5)	7 (2.6)	NR
WHO [23]	Nicaragua	171	Chronic (2015–2016)	40 (23.4)	33 (19.3)	18 (10.5)	0 (0)	NR
WHO [23]	Argentina	294	Chronic (2014–2016)	41 (13.8)	33 (10.9)	10 (3.7)	6 (1.9)	NR
WHO [23]	Brazil	1391	Chronic (2013–2016)	137 (9.8)	94 (6.8)	50 (3.6)	13 (0.9)	NR
WHO [23]	Colombia	192	Chronic (2016)	19 (9.9)	12 (6.3)	7 (3.6)	0 (0)	NR
WHO [23]	Myanmar	327	Chronic (2016)	21 (5.4)	16 (3.9)	5 (1.4)	1 (0.2)	NR
Metanalysis, Gupta et al. [5]	South Africa	11 855	Chronic (estimates for 2016)	11% [7.5–15.9]	10.7%^b^ [8.4–13.7]	2.2^b^ [1.2–3.8]	NR	NR
Metanalysis, Gupta et al. [5]	Eastern Africa	7169	Chronic (estimates for 2016)	10.1% [5.1–19.4]	10.1%^b^ [8.2–12.4]	3.2%^b^ [3.3–8.5]	NR	NR
Metanalysis, Gupta et al. [5]	Western and Central Africa	4924	Chronic (estimates for 2016)	7.2% [2.9–16.5]	5.3%^b^ [3.3–8.5]	3.7%^b^ [2.0–6.5]	NR	NR
Metanalysis, Gupta et al. [5]	Latin America and Caribbean	16 008	Chronic (estimates for 2016)	9.4% [6.2–12.4]	8.8%^b^ [6.2–12.4]	4.1%^b^ [2.5–6.5]	NR	NR
Metanalysis, Gupta et al. [5]	Asia	16 088	Chronic (estimates for 2016)	3.2% [1.8–5.6]	4%^b^ [2.1–6.7]	1.5^b^ [0.5–3.5]	NR	NR

Abbreviations: InSTI, integrase strand transfer inhibitor; NNRTI, nonnucleoside reverse transcriptase inhibitor; NR, not reported; nRTI, nucleoside reverse transcriptase inhibitor; PDR, pretreatment drug resistance; PI, protease inhibitor; WHO, World Health Organization.

^a^Values are provided as either No. (%) or % [confidence interval].

^b^Data published in supplementary appendix on page 18 of Gupta et al. [5].

A meta-analysis that included 19 studies, representing 2617 children from 13 countries, found a prevalence of PDR of 42.7% in children exposed to ART as treatment for prevention of mother-to-child transmission (PMTCT) and 12.7% among children not exposed to PMTCT treatment. The prevalence of TDR in children not exposed rose from 0% in 2004 to 26.8% in 2013. NNRTI mutations were found in 32.4% of PMTCT treatment–exposed and in 9.7% of PMTCT treatment–unexposed children [24]. Representative data on PDR prevalences are limited in LMIC, and thus prevalences are likely underestimated.

### Factors Associated With Transmission

In Switzerland and the United Kingdom, the major source of TDR in men who have sex with men are ART-naïve, HIV-1–infected individuals [18, 25]. This unexpected finding is due to some DRMs with low fitness cost; fitness cost is defined as reduced ability of a virus to replicate in the absence of the drug. For example, both the DRM L90M, conferring resistance to PIs, and the DRM K103N, conferring resistance to NNRTIs, have low fitness costs and persist for prolonged periods after transmission; they are prevalent in this population [20, 26]. Generally, drug-resistant viruses will be gradually replaced by fitter viruses in the absence of drug pressure; the rate of replacement depends on the degree of the fitness cost. The sources of most transmissions are individuals with acute or recent infections [27–29]. Recommendations to treat all HIV-1–infected individuals, including patients diagnosed early after infection, have been inadequately implemented to optimally reduce new transmissions [2, 30–35].

### Transmission of Minority Variants Harboring Drug-resistant Mutations

Recommendations are given in Appendix Box 2. In 70% to 90% of acute HIV-1 infections, single-virus variants are detected. However, in 10% to 30%, minority variants are transmitted, often at very low frequencies [36–38]. Minority variants harboring DRMs have been identified in drug-naive and primary infection populations [39–42].

Minority variants harboring DRMs have not been shown to negatively affect treatment responses in acutely-infected patients [41]; however, in some studies they modestly reduced treatment success in chronically-infected drug-naive patients receiving first-generation NNRTI (efavirenz or nevirapine)-based treatment [39, 42, 43]. More information on minority variants and the relevance of cut-offs to detect different frequencies of variants harboring DRMs is available in the Supplementary Data.

## EMERGENCE OF RESISTANCE WITH LOW-LEVEL VIREMIA

Recommendations are given in Appendix Box 3. Suppression of HIV ribonucleic acid (RNA) below limits of quantification is the objective of ART [44]. Detectable viremia during ART between the limits of the assay quantification (20 to 50 copies/mL) and 1000 copies/mL is generally referred to as a low-level viremia (LLV). An LLV that is transient and only observed in a single measurement followed by subsequent undetectable viral load is termed a viral blip. A detectable viremia below the limit of quantification is referred to as a residual viremia or very LLV. The source and clinical relevance of very LLV and viral blips are unknown. LLV and blips may reflect technical variability or real biologic processes. A proposed mechanism is the release of a virus from activated, latently-infected cells that, in the presence of ART, does not result in active rounds of virus replication. Low levels of ongoing virus replication due to poor adherence or insufficient drug penetration in certain tissues and anatomical compartments may also occur [45–50].

Drug resistance can emerge in patients with an HIV-1 RNA below 1000 copies/mL. Studies have utilized different thresholds for LLV and different criteria for resistance; however, each confirmed that drug resistance can emerge at low levels of HIV replication, in the range of 50 to 200 copies/mL, with increased risk at higher levels in the presence of the selective pressure of ART [51–53].

Although resistance assay kits are only approved by the Food and Drug Administration for HIV RNA levels above 1000 copies/mL, resistance testing is feasible at lower ranges of viremia [44, 51, 53–55]. During polymerase chain reaction amplification, more plasma can be used to increase the amount of HIV RNA extracted. The Supplementary Data summarizes existing data on the relevance of LLV and blips in treated individuals in more detail.

## EFFECT OF SUBTYPE ON HIV-1 DRUG RESISTANCE

Recommendations are given in Appendix Box 4. HIV-1 group M viruses have evolved into numerous subtypes and circulating recombinant forms, differing from each other by approximately 12% in HIV-1 *pol*. Subtype B viruses account for about 10% overall, but have been disproportionately studied, as they are the predominant subtype in North America and Europe. As ART has expanded globally, the effect of subtype on HIV-1 drug resistance has received increasing attention.

Virtually all amino acid differences between subtypes are polymorphisms: variants that occur commonly in the absence of therapy and have little, if any, phenotypic or clinical effect on antiretroviral drugs (ARVs). Thus, most studies suggest that individual ARVs and standard ART regimens are similarly active, regardless of subtype [56–63]. Increased risk of virologic failure has been associated with particular subtypes, but most studies are confounded by geographic location, treatment facilities, and clinical, social, and economic statuses that could affect outcome differences among individuals infected with different subtypes [64–67].

HIV-2 infects more than 1 million persons, most of whom reside in or have emigrated from West Africa [68]. It differs from HIV-1 by more than 50% of its genome. HIV-2 is intrinsically resistant to NNRTIs and is variably susceptible to PIs [69, 70]. The potential impact of different subtypes on ART outcomes is discussed in the Supplementary Data.

## METHODS FOR HIV-1 RESISTANCE TESTING

Recommendations are given in Appendix Box 5.

### Resistance Test Options

In most situations, genotypic testing for resistance is the test of choice because it is faster, less expensive, and sufficient to predict drug susceptibility. Phenotypic testing is more expensive, technically demanding, and requires a highly-specialized laboratory infrastructure, but is recommended in certain situations. Methodologic aspects and challenges of resistance testing are described in the Supplementary Data.

The bulk of evidence associating resistance with clinical outcomes has been generated with sequencing of plasma HIV RNA. For individuals with LLV (ie, <200 copies/mL), sequencing of peripheral blood mononuclear cell (PBMC) DNA is technically feasible and more likely to provide an HIV genotype than plasma. However, PBMCs contain HIV DNA that has been archived throughout the patient’s infection, and may be discordant with plasma testing. Clinicians should judge results from PBMC testing with caution [71, 72]. More detailed discussion on proviral DNA sequencing is provided in the Supplementary Data.

### Recommended Genomic Regions for Sequencing

To determine what new regimen to use for patients in whom ART is failing, the protease and the first half of the reverse transcriptase (up to at least nucleotide 215) should be sequenced. If an InSTI-containing treatment has failed, integrase should be sequenced. Although of interest for better understanding of patterns at time of failure, baseline InSTI resistance testing is currently not cost-effective, as transmitted InSTI resistance is infrequent [73]. Baseline InSTI resistance testing should be considered, however, in select patients with evidence of TDR, such as those with nRTI- or multi-class resistances. In such patients, the risk of also having transmitted InSTI resistance is likely to be higher than in patients without TDR, and the consequences of virologic failure on an InSTI-containing initial regimen may be more severe.

Sequencing of other regions (C-terminus of reverse transcriptase, group-specific antigen) or even a near-full length of HIV-1 might be useful in research settings [74]. Sequencing of the third variable loop (V3) of the envelope glycoprotein, gp120, can determine whether a virus is R5 tropic, and thus might respond to inclusion of a chemokine receptor 5 (CCR5) antagonist in ART. Genotypic tropism testing performance might be comparable to phenotypic tropism assays, particularly when NGS is used [75–78]. However, testing for genotypic tropism from the PBMC compartment is less accurate than plasma testing [75]. A more detailed discussion of entry inhibitor resistance and novel drug formulations is available in the Supplementary Data.

### Genotypic Resistance Test Interpretation

Genotypic test results require an interpretation, because there are many DRMs that often arise in complex patterns and cause varying levels of reduced drug susceptibilities [79, 80]. Genotypic test interpretation systems are either rule-based or machine-learning systems. Rule-based systems require a knowledge base and a set of derived rules. The knowledge base usually comprises studies of whether a drug selects a DRM in vitro or in patients, whether a DRM reduces drug susceptibility in site-directed mutants or clinical isolates, and whether a DRM is associated with reduced virologic response to a regimen containing a specific ARV. Virologic response studies have usually been performed in the context of clinical trials [81]. Such studies have assessed the effect of nRTI-associated DRMs on virologic responses to regimens containing abacavir or tenofovir disoproxil fumarate [82, 83]; PI-associated DRMs on responses to regimens containing lopinavir/ritonavir and darunavir/ritonavir [84, 85]; NNRTI-associated DRMs on responses to regimens containing etravirine [86]; and InSTI-associated DRMs on responses to regimens containing dolutegravir [87].

Rule-based systems are more commonly used for interpretation, because they consider diverse forms of data and incorporate expert opinions [80, 88–90]. These systems are reproducible, transparent, and educational, but subjective. Well-described rule-based systems include those from the French National Agency for Research on AIDS and Viral Hepatitis, Rega, HIV Genotypic Resistance-Algorithm Deutschland, and the Stanford HIV Drug Resistance Database [80, 91, 92]. Although these systems may produce somewhat different estimates of drug resistance for the same drug, their predictive ability generally has been similar [88, 89, 93]. An online system for interpreting HIV-2 sequences has also been developed [94].

Machine-learning systems use datasets containing large amounts of data; for example, correlating DRMs in a sequence with reduced susceptibility [95–97] or with virologic response to a new treatment regimen [98, 99]. With sufficiently large numbers of such correlations, these systems can use genotypic data to predict fold-reductions in susceptibility [95, 96] or the likelihood of virologic suppression with a new regimen [97–99].

### Interpretation of Phenotypic Resistance Tests

Phenotypic test results require interpretation, because the clinical significance of fold-change reductions in susceptibility differs among drugs [98, 100]. Interpretation of these results usually requires studies that indicate cutoff values for each drug: the fold-reduction in drug susceptibility that exceeds the uppermost values for wild-type viruses (biologic cutoff) [100]; the lowest fold-reduction in susceptibility that indicates reduced likelihood of responding to therapy with a drug (lower clinical cutoff); and the lowest fold-reduction in susceptibility that indicates a drug will likely be completely inactive (upper clinical cutoff). Clinical cutoff values for an ARV are assay-dependent, and therefore need to be established for each phenotypic assay used in a clinical setting [98, 101].

### Limitations of Drug Resistance Interpretation Systems

Genotypic and phenotypic test interpretations cannot provide specific treatment recommendations, because they do not integrate all data required for therapy selection, such as treatment history, previous resistance test results, minority variants harboring DRM archived in the latent reservoirs, plasma HIV-1 RNA level, CD4+ cell count, pharmacologic interactions, hepatic and renal status, or the likelihood of adherence. Interpretation systems vary in how they account for differences in potencies of different ARVs, and do not incorporate fundamental principles of how specific regimens should be constructed. Therefore, clinicians must either have a sound understanding of the principles of therapy to optimally use results of drug resistance tests or have access to expertise [33, 35]. An extended discussion on interpretation systems is available in the Supplementary Data.

## CLINICAL APPLICATIONS AND RECOMMENDATIONS

Table 4 summarizes in whom and when genotypic resistance testing should be performed. With the recent recommendations to initiate therapy with an InSTI [33], the current likelihood of a compromising DRM is low. Regardless, even if treatment is initiated quickly, adjustments can be made within days if test results so indicate. The HIV-1 RNA threshold of 200 copies/mL is a technical limit; that is, the minimum copy number at which the likelihood of obtaining a test result is above 70% [53, 55, 102]. HIV genotyping below 200 copies/mL may produce clinically meaningful data, but the chance of obtaining an interpretable genotype may be too low for routine clinical use [53, 55, 102]. Access to drug resistance testing may be limited by economic constraints or local technical capacity

**Table 4.** Recommendations for Resistance Testing in Clinical Practice: Who and When to Test

Recommendations	When to Test	Gene to be Sequenced			Strength/ Evidence	Comments
		Protease	Reverse Transcriptase^a^	Integrase		
HIV resistance testing is recommended for all individuals with HIV infection:						
• who are newly diagnosed and presumably ART-naïve;	As soon as an individual is diagnosed with HIV-1 infection. In any case, before ART is started.	Yes	Yes	(Yes)^b^	AIIa	To detect transmitted RAM. Early testing increases the chances of detecting TDR before mutations are potentially replaced by wild-type virus (particularly relevant for high–fitness cost mutations, eg, M184V, K65R, T215Y, and others). Many resistance mutations can still be detected even years after infection; in particular, low–fitness cost mutations (eg, K103N, L90M, etc). InSTI TDR is currently rare.
• who are on antiretroviral treatment and have plasma HIV RNA that is rising to above 200 copies/mL by confirmed measurements after they have been suppressed to below 50 copies/mL;	Preferably while on failing ART.	Yes	Yes	Yes	AIIa	To detect acquired drug resistance in patients who initially responded to ART and, later on, failed. InSTI RAM should be tested in all treatment failures.
• who have not achieved full virus suppression after initiating ART;	≥6 months after ART initiation.	Yes	Yes	Yes	AIIa	To detect acquired drug resistance in patients who did not achieve successful viral suppression to antiretroviral treatment. InSTI RAM should be tested in all treatment failures.
• who have interrupted ART containing an NNRTI with a long half-life (eg, efavirenz); or	As soon as virus rebounds above 500 HIV-RNA copies/ mL, respectively, before re-initiation of ART.	Yes	Yes	Yes	AIIa	Treatment interruption of such regimens can lead to virtual monotherapy with rapid emergence of resistance.
• who have a significant increase in viral load in a drug-naive individual not on treatment.^c^	After confirmation of increase in plasma viremia.	Yes	Yes	(Yes)^b^	AIII	Superinfection with drug-resistant virus may occur (consider also tropism testing, because a switch from CCR5- to CXCR4 tropic virus may have occurred).

Abbreviations: ART, antiretroviral therapy; HIV, human immunodeficiency virus; InSTI, integrase strand transfer inhibitor; NNRTI, nonnucleoside reverse transcriptase inhibitor; RAM, resistance-associated mutation; RNA, ribonucleic acid; TDR, transmitted drug resistance.

^a^Sequencing of first half of the reverse transcriptase up to at least nucleotide 215 is sufficient.

^b^Currently, evidence of InSTI TDR is rare. Thus “Yes” in brackets (YES) means that InSTI testing should be considered if certain circumstances are given (see comments).

^c^Increase of plasma viremia of >0.5 log_10_ within approximately 3–6 months that is confirmed by a second HIV-1 RNA measurement.

## USE OF GENETIC SEQUENCES FOR OTHER PURPOSES

With routine use of resistance testing for clinical purposes in the late 1990s [103], it became possible to establish large sequence databases [20, 104]. Linking sequences to clinical and epidemiologic data provides a public health infrastructure to monitor transmission of drug resistance and the risk conferred by the emergence of resistance on therapy. These approaches have also defined and validated clinical interpretations of drug resistance, such as those described earlier.

Large HIV genetic databases have also facilitated the application of increasingly sophisticated phylogenetic-based analyses to assess dynamics of virus spread, taking advantage of the inherent variation between sampled viral sequences [105]. This approach has uncovered key characteristics of the epidemic, particularly where the density of sampling (eg, the proportion of infected individuals represented by viral sequence) has been high. Use of large-scale viral sequences for purposes other than resistance testing is described in the Supplementary Data.

## SUMMARY AND FUTURE DIRECTIONS

Despite the unprecedented global rollout and success of ART, drug resistance continues to emerge with treatment failure, and TDR persists. Divergent patterns of resistance between resource-rich and -limited settings have evolved. In the former, resistance testing, viral load monitoring, and access to care and to ART have resulted in a continuing decrease of acquired drug resistance and stable rates of TDR. In contrast, in LMIC, limited availability of drugs and inadequate monitoring of and acting on viral load and drug resistance has contributed to a steep increase in drug resistance. New strategies, such as starting with regimens with greater potency and genetic barriers (eg, with InSTIs), providing increased options for second- and third-line regimens, implementing viral load testing to identify virologic failures early, and raising capacities for resistance testing at baseline and after treatment failure are prerequisites to secure long-term global success of ART [106].

Since the last report [8], recommendations to initiate ART have fundamentally changed; all individuals with HIV infections should be treated as early as possible after infection, regardless of CD4+ count [2, 33, 35, 107–111]. Concerns that large-scale earlier treatment initiation would give rise to more resistance has not been confirmed in resource-rich settings; in fact, resistance emergence decreases with earlier treatment initiation [1, 112, 113].

This report is primarily written for settings that have access to resistance testing for HIV patient management, whereas most people with HIV live in LMIC, where most HIV drug resistance testing is applied to epidemiologic monitoring. Available data on prevalence and TDR or PDR in LMIC are limited and delayed, thus often underestimating those numbers.

In conclusion, testing for HIV drug resistance, and the appreciation of its role, is crucial to the prevention and management of failure of ART.

## Supplementary Data

Supplementary materials are available at *Clinical Infectious Diseases* online. Consisting of data provided by the authors to benefit the reader, the posted materials are not copyedited and are the sole responsibility of the authors, so questions or comments should be addressed to the corresponding author.

## Supplementary Material

Supplementary Material

## Notes

The panel designed and conducted the work; collected, managed, analyzed, and interpreted the data; and prepared, reviewed, and approved the manuscript.

***Acknowledgments.*** International Antiviral Society-USA determined the need to update recommendations, vetted and appointed panel members, and provided administrative support and oversight. At least 1 member of the volunteer Board of Directors serves on the panel. We thank Dr Victoria Johnson for insightful comments to the outline of this report, Michelle Valderama for valuable administrative support, and Mayra Rodriguez for assistance with the literature searches.

***Financial support.*** This work was supported by the International Antiviral Society–USA, a 501(c)(3) not-for-profit organization whose mission is to provide balanced and relevant information and education for practitioners involved in HIV disease management and research. No commercial or government funding was used to support the work.

***Potential conflicts of interest.*** H. F. G. has received grants from the Swiss National Science Foundation; Swiss HIV Cohort Study; Yvonne Jacob Foundation; University of Zurich’s Clinical Research Priority Program, viral disease; Zurich Primary HIV Infection; Systems.X; National Institutes of Health; Gilead Sciences; and Roche; and personal fees from Merck, Gilead Sciences, Teva, and Sandoz for consultancy. V. C. has served as an advisor or consultant to and has received research grants from Bristol-Myers Squibb; Gilead Sciences, Inc.; Johnson and Johnson; and ViiV Healthcare; and is a founder of SkinDermic Pharma. R. P. has received research grants from ViiV Healthcare and Merck. R. W. S. has received grants from Gilead Sciences; Janssen Pharmaceuticals; Vela Diagnostics; Bristol-Myers Squibb; and Merck; and personal fees from Abbott Diagnostics and ViiV Healthcare. A. M. W. has served on advisory boards for CLJI Worldwide; Gilead Sciences, Inc.; Merck; Janssen Pharmaceuticals; and ViiV Healthcare; has received grants from Janssen Pharmaceuticals; Gilead Sciences, Inc.; and ViiV Healthcare; and has received support from Virology Education (Conference organizer). D. M. J. has nothing to disclose. D. D. R. has been a consultant to Antiva Biosciences; Chimerix; Gilead Sciences, Inc.; and Monogram Biosciences, Inc. In the prior 5 years, International Antiviral Society-USA has received grants for several continuing medical education activities that are pooled (ie, no single company supports a single effort) from Abbvie; Bristol-Myers Squibb; Gilead Sciences; Janssen Therapeutics; Merck; Salix Pharmaceuticals, Inc.; Vertex Pharmaceuticals, Inc.; and ViiV Healthcare. All other authors report no potential conflicts. All authors have submitted the ICMJE Form for Disclosure of Potential Conflicts of Interest. Conflicts that the editors consider relevant to the content of the manuscript have been disclosed.

## APPENDIX

Box 1. Recommendations for Prevalence of Transmitted or Pretreatment Drug Resistance in Resource-rich and -limited Settings (As Available Resources Allow)
•Resistance testing in drug-naive individuals is recommended at the time of diagnosis to detect potential transmitted drug resistance (TDR; evidence rating AIIa).
•TDR and pretreatment drug resistance should be monitored on a country level, accounting for different transmission groups (evidence rating AIIa).
•Resistance testing is recommended for perinatally-infected children, particularly those whose mothers received prevention of mother-to-child transmission treatment (evidence rating AIIa).

Box 2. Recommendations for Transmission of Minority Variants Harboring Drug-resistant Mutations
•Drug resistance testing to detect minority variants is not currently recommended outside of research settings, but may be considered for nonnucleoside analogue reverse transcriptase inhibitors (NNRTIs; evidence rating AIIa).

Box 3. Recommendations for detection of Resistance With Low-level Viremia •Samples with a blip exceeding 200 human immunodeficiency virus (HIV)-1 ribonucleic acid (RNA) copies/mL should be considered for resistance testing, if available (evidence rating CIII).
•Resistance testing is recommended in patients experiencing low-level viremia above 200 copies/mL (evidence rating AIIa) viremia.
•To avoid biases during polymerase chain reaction amplification, more plasma can be used to increase the amount of HIV RNA extracted (evidence rating BIII).

Box 4. Recommendations for Effect of Subtype on HIV-1 Drug Resistance
•HIV-1 subtype need not be a consideration regarding HIV drug resistance in selecting antiretroviral therapy (ART) regimens with nucleoside analogue reverse transcriptase inhibitors (nRTIs), NNRTIs, protease inhibitors (PIs), and integrase strand transfer inhibitors (InSTIs; evidence rating AIII).
•For HIV-2, NNRTIs should be avoided regardless of resistance testing, whereas PIs should be used only under the supervision of a physician experienced at using this drug class for treating HIV-2 (evidence rating AIIa).

Box 5. Recommendations for Methods for HIV-1 Resistance Testing
•As a first choice, genotypic resistance testing is recommended (evidence rating AIIa).
•Phenotypic resistance testing is recommended, in certain situations:

1. to evaluate HIV susceptibility to new and investigational drugs when drug-resistant mutation patterns have not been fully established (evidence rating AIIa);
2. when genotypic test results are too complex to interpret (evidence rating CIII); or
3. when ART options are highly limited and, as a result, salvage ART must rely on residual susceptibilities to different drugs that are difficult to predict from genotypic data (evidence rating CIII).

•The recommended compartment for drug resistance testing is plasma (evidence rating AII).
•Inclusion of the protease and first half of the reverse transcriptase (up to at least nucleotide 215) is recommended for all genotypic testing (evidence rating BIII).
•Routine InSTI resistance testing in drug-naive individuals is currently not recommended (BIII).
•Baseline InSTI resistance testing is recommended in select patients with evidence of TDR, such as those with nRTI- or multi-class resistance (evidence rating AIII).
•Monitoring of TDR/pretreatment drug resistance to InSTI in selected sites in resource-rich settings and low- and middle-income countries is recommended (evidence rating AIII).
•Sequencing of other regions (C-terminus of reverse transcriptase, *gag*) or even a near full-length of HIV-1 is not recommended for routine clinical management (evidence rating AIIa).
•Genotypic tropism testing is recommended if a CCR5 antagonist is considered for treatment (evidence rating BIIa).
•Peripheral blood mononuclear cell genotypic resistance testing is recommended in patients with low-level viremia or in patients who are virologically suppressed (evidence rating AIII).
