# Supplementary material for: Human Immunodeficiency Virus Drug Resistance: 2018 Recommendations of the International Antiviral Society–USA Panel
Source: Clin Infect Dis. 2018 Jul 20;68(2):177–87. doi: 10.1093/cid/ciy463 (PMC6321850; doi:10.1093/cid/ciy463)
Supplement: Supplementary Material [file ciy463_suppl_supplementary_materials.docx]

**Supplementary On-line Content**

**eMethods:** Recommendations Development Process

**eBox 1.** Volunteer IAS–USA Board of Directors, December 2017

**eBox 2.** IAS–USA Antiretroviral Drug Resistance Testing Recommendations Panel

**eBox 3.** Working Sections of the IAS–USA Antiretroviral Drug Resistance Testing Recommendations Panel

**eTable 1.** Summary of Evidence Collection

**eTable 2.** Search Terms Used and Results of Embase and PubMed Literature Searches

**eTable 3.** Strength of Recommendation and Quality of Evidence Rating Scale

This supplementary material has been provided to give readers additional information about the process and the work.

Extended discussion of specific topics according to main manuscript

Here some sections of the main manuscript are discussed in more depth including additional references.

**eBox 4.** Sanger and Next-Generation Sequencing (NGS) Methods

**eMethods: Recommendations Development Process**

**I. Brief Summary**

The recommendations for antiretroviral drug resistance testing in adults with HIV infection were developed by an international panel of experts in HIV research and patient care. The initial Panel was established in 1998 by the International Antiviral Society–USA (IAS–USA);^1^ members are selected by the IAS–USA Board of Directors and vetted by the organization for suitability for the panel. Panel members serve in a volunteer (uncompensated) capacity and do not participate in industry promotional activities such as speakers’ bureaus, lectures, or other marketing activities during their tenure on the panel. Members of the current panel convened in person in February 2017 and by conference calls throughout 2017. The chair (Huldrych F. Günthard, MD) oversees the discussions of the process and evidence review and manuscript development, and guides the group to consensus. Section leaders (**eBox 3**) were appointed to evaluate evidence and summarize panel discussions for each section. Prior to selection of the section leaders, panel members declared their financial relationships with commercial concerns, discussed potential conflicts of interest (COIs), and recused themselves from serving as section leaders as necessary.

Evidence considered for updating the recommendations was limited to data published in the scientific literature, presented at major peer-reviewed scientific conferences, or released as safety reports by regulatory agencies or data safety and monitoring boards, since the last update in 2008 through September 2017. (Section VI).

Drug resistance testing has become widespread and accepted as an important adjunct to patient management. Recommendations were made by full-panel consensus and rated according to the strength of the recommendation and the quality of the supporting evidence (Manuscript Table 1). For areas in which recommendations have not changed substantially or no or few new data are available, the reader is referred to the previous report.^2^

**II. Detailed Summary**

**a. Background**

The IAS–USA Antiretroviral Drug Resistance Testing Recommendations Panel was first convened by IAS-USA in 1997 to develop evidence-based recommendations for the assessment of HIV-1 drug susceptibility and the management of drug-resistant HIV-1 infection in clinical practices in the developed world. Panel members are not compensated, and there is a process for panel member rotation. Updated reports are initiated when enough new published presented information in the field accumulated to warrant revising previous recommendations. The panel has published recommendations for HIV-1 drug resistance testing in HIV-1–infected adults in 1998, 2000, 2003, and 2008.

**b. The IAS–USA and Its Role in the Recommendations**

The IAS–USA is a 501(c)(3) not-for-profit, mission-based, nonmembership, educational organization that was established in 1992. The mission of the IAS–USA is to improve the prevention, treatment, care, and quality of life for people with or at risk of HIV, hepatitis C virus (HCV), or other viral infections through high-quality, relevant, balanced, and needs-oriented education and information for practitioners and scientists who are actively involved in medical care and research. The IAS–USA delivers annual continuing medical education (CME) programs on HIV and HCV that include live courses; live intensive, interactive workshops; live webinars; online interactive activities in the series *Cases on the Web* (*COW*); and the peer-reviewed, indexed journal *Topics in Antiviral Medicine*™. In addition, IAS–USA manages and serves as the CME sponsor for the annual Conference on Retroviruses and Opportunistic Infections (CROI), a research conference.

IAS–USA has sponsored the development of evidence-based recommendations for viral load monitoring, antiretroviral therapy, HIV drug resistance testing, cytomegalovirus (CMV) infection, and the metabolic complications of antiretroviral therapy, all of which are published in the medical literature.^1-20^

The volunteer members of the IAS–USA Board of Directors (**eBox 1**) oversee the development of the information and educational programs and are not compensated for their roles in oversight and governance of the organization.

IAS–USA funding comes from a variety of sources. The largest single source of revenue is conference and CME participant registration fees. Two large national CME efforts (one on HIV and another on HCV) invite funding in the form of educational grants from industry. Per IAS–USA policy, any effort that uses commercial grants must receive grants from several companies with competing products. Funds are pooled and distributed to activities within the effort at the sole discretion of the IAS–USA. Grantors have no input into any activity, including its content, development, or selection of topics or speaker(s). Grantors are listed in each activity as applicable. Other funding sources include grants and subcontracts from government agencies, private donations, and gifts-in-kind from local community businesses and individuals.

IAS-USA is accredited with commendation by the Accreditation Council for Continuing Medical Education (ACCME) to provide continuing medical education (CME) for physicians.

The development of the Antiretroviral Drug Resistance Testing Recommendations is supported and funded by the IAS–USA. The IAS–USA determined the need for updated recommendations; selected panel members based on expertise in research and care to represent developed-world settings affected by HIV disease; determined the most appropriate way in which to dissemble the information (eg, publication in a medical journal rather than publication in the IAS–USA journal, web publication, etc); and provided administrative oversight and financial support.

The Panel itself is responsible for proposing the design and conduct of the work; collection, management, analysis, and interpretation of the data; and preparation, review, and approval of the manuscript. IAS–USA provided staff support for administrative management, oversight of literature searches and editorial and production assistance. At least one member of the Board serves in each panel to ensure continuing with the IAS–USA mission.

**c. Identifying and Screening Panel Members**

The panel was initially appointed in 1997, and members have rotated periodically since then. In evaluating potential participants for the Panel, the IAS–USA Board considered individuals who 1) are recognized as authorities in HIV treatment research and clinical care, 2) have appointments in major medical teaching or research institutions, 3) have a demonstrated ability to review and evaluate evidence in an effort to provide useful recommendations in the field, 4) meet the IAS–USA COI and financial relationship criteria for participation (see below and [www.iasusa.org](http://www.iasusa.org)), and 5) have the ability to work in a collaborative consensus process. In addition, the Board emphasized the need for an international, developed world perspective.

Like the IAS–USA Board of Directors, participants in IAS–USA panels are volunteers and receive no financial compensation for their panel participation. In joining the Panel, members agree to commit substantial time to the effort necessary for evidence review and for participation in the consensus process.

**d. COI Management**

It is the policy of IAS–USA to ensure balance, independence, objectivity, and scientific rigor in all its activities. All parties with control over the content of IAS–USA activities are required to disclose to the organization and activity audience any financial interest or other relationship with the manufacturer(s) of any commercial product(s) or provider(s) of commercial services with interests discussed in the activity (eg, presentation, article, etc) within at least the past 12 months. Financial interests or other relationships can include receipt of grants or research support, status as employee or consultant, stock or options holder, paid lecturer, paid lecturer, writer, or author, or member of speakers bureau, of the party or of his or her spouse or partner. The ACCME defines a financial interest as an interest of any dollar amount. Part of the IAS–USA policies to ensure the integrity of its activities is the policy to separate commercial promotion from core IAS–USA educational and informational activities. Individuals who conduct marketing or promotional activities for commercial firms may not contribute to core IAS–USA programs. A marketing or promotional activity includes any activity in which the commercial entity controls key elements, such as speaker or topic selection, which could be used to serve the entity’s commercial interests (eg, speakers bureaus, advertorials, etc). Individuals may not participate in most IAS–USA programs for 12 months after functioning in a promotional or marketing effort for a commercial firm. A notable exception to the separation policy is the annual Conference on Retroviruses and Opportunistic Infections (CROI) which allows research and symposia presentations by individuals with some of such relationships (including employment) because of its large focus on the presentations on original research, if their research or work passes rigorous peer review). Panel members who meet general criteria and are appointed, agree not to participate in any promotional activity on behalf of a pharmaceutical or medical device company (eg, serve on a speaker bureau, as a paid lecturer, or a similar contribution) while a member of the panel. Any conforming financial relationships with commercial entities that still may represent a real or potential COIs will be resolved so that they do not influence the content of the recommendations. Prior to selection of the section teams and leaders, panel members declared their financial relationships with commercial concerns, discussed potential COIs, and recused themselves from serving as section leaders or team members accordingly.

**III. The IAS–USA Antiretroviral Drug Resistance Testing Recommendations Panel**

The members of the IAS–USA Antiretroviral Drug Resistance Testing Recommendations Panel are listed in **eBox 2**. The Panel convened in person in February 2017, and regularly by conference call. The chair oversees the discussions of the process and evidence review and manuscript development, and guides the group to consensus. Section leaders and teams were appointed to evaluate evidence and summarize panel discussions for each section.

**a. Method**

This review and the recommendations were developed by an international panel of experts in HIV research, virology, and drug resistance appointed by the International Antiviral (formerly AIDS) Society-USA (IAS-USA). Members were vetted for their expertise and ability to work toward consensus. Financial relationships with commercial enterprises were reviewed before appointment to the panel. Individuals who engage in commercial promotional activities (eg, on speaker’s bureaus or serving as a product spokesperson) were not eligible to participate, but research support or scientific consultancy were acceptable relationships. Panel members were not compensated for their service on the panel. The panel first convened in person for this updated report in March 2017 to discuss changes since the last report^9^ and to agree on an outline and issues to be addressed. The full panel met regularly thereafter via conference calls.

Evidence considered for updating the recommendations was limited to data published in the scientific literature, presented at major peer-reviewed scientific conferences, or released as safety reports by regulatory agencies or product manufacturers, since the last update in 2008 through September 2017. Panel member knowledge of existing data served as the primary source of relevant evidence. Literature searches in PubMed and Embase supplemented or validated evidence considered. Initial literature searches by subcategory in this report were conducted in PubMed and Embase. The initial results of are summarized in **eTable 2**. Each panel member (and particularly the section leads) reviewed the respective results and conducted more focused searches as needed. Panel members and leads reviewed recent scientific meetings to identify relevant data to the section topic or report overall.

The panel was divided into teams (each with a lead as noted) to develop each section and propose the recommendations, evidence, and evidence ratings. All panelists reviewed the interim drafts and recommendations. Full recommendations were made by full panel consensus (ie, agreement) and were rated according to the strength of the recommendations and quality of the evidence **(eTable 3)**.

**IV. Rating the Recommendations**

The Panel is divided by topic into working sections, each with a section leader. These sections are responsible for reviewing and screening evidence, developing preliminary recommendations, and presenting these to the full Panel for discussion, identification of further evidence, and consensus.

The selected rating system (**see eTable 2**) combines 2 ratings for each recommendation. One rates the strength of the recommendation (strong, moderate, or limited support) and the other rates the quality of the evidence (ranging from Ia, based on evidence from 1 or more randomized controlled clinical trial[s] published in the peer-reviewed literature, to III, based on the Panel’s analysis of the accumulated available evidence [**eTable 3**]).^21^

**V. Content of the Recommendations**

The Panel agreed on the purpose, audience, and scope of these recommendations and on 8 main content sections (and subsections).

Content Sections and Section Leads/Teams:

1. Introduction – Huldrych Günthard, MD, and Douglas D. Richman, MD
2. Methods for Resistance Testing – Roger Paredes, MD, PhD
3. Transmission, Origin, and Epidemiology of Drug Resistance – Huldrych Günthard,, MD
4. Selection of Resistance with Low-Level Viremia – Annmarie Wensing, MD, PhD
5. Effects of Subtype on HIV-1 Drug Resistance – Robert Shafer, MD
6. Drug Resistance Test Interpretation – Robert Shafer, MD, and Vincent Calvez, MD
7. Clinical Applications and Recommendations – Roger Paredes, MD, PhD
8. Use of HIV Drug Resistance Genetic Sequences for Other Purposes – Deenan Pillay, MD, PhD
9. Future Directions – Huldrych Günthard, MD, and Douglas D. Richman, MD

Panel members were assigned to content sections based on their expertise and section leaders were appointed (**eBox 3**). Douglas D. Richman, MD, Panel Co-Chair, reviewed the literature search results and identified relevant publications, and also reviewed the entire manuscript.

From February 2017 on, sections met in person and by conference call and e-mail exchange. Initial discussions were used to develop detailed Section outlines, and assign participants to draft subsections. The full Panel reviewed sections and the final manuscript.

**VI. Evidence Collection and Literature Searches**

Panel members were selected based on their active work in the field of HIV drug resistance and care, and detailed knowledge of available evidence (published and presented at major scientific conferences).

Keywords were provided by all panel members. Literature searches in PubMed and Embase were then conducted by an expert in systematic reviews who works with Douglas D. Richman, MD, at the University of California San Diego. One of the other panel members, Robert Shafer, MD, also conducted 2 searches. Results from all searches are shown in **eTable 1**. The initial literature search provided data available since the 2008 publication of the recommendations through August 2017 Approximately 333 references were ultimately identified. The panel members, all noted virologists, drew upon their expertise to identify relevant abstracts, published papers, and other reports for consideration.

**eBox 1. Volunteer IAS–USA Board of Directors, December 2017**

Paul A. Volberding, MD, Chair

Professor of Medicine

Co-Director, Center for AIDS Research

Director, AIDS Research Institute

University of California San Francisco

San Francisco, California

Constance A. Benson, MD, MPH

Professor of Medicine

I.D. Training Program Director

Director, Antiviral Research Center (AVRC)

PI/Director, HIV/AIDS Clinical Trials Unit

University of California San Diego
San Diego, California

Peter C. Cassat, JD

Vice President and General Counsel

AutoTrader.com

Atlanta, Georgia

Judith S. Currier, MD

Professor of Medicine

Chief, Division of Infectious Disease

Associate Director, Clinical AIDS

Research and Education Center

David Geffen School of Medicine

University of California Los Angeles

Los Angeles, California

Carlos del Rio, MD

Hubert Professor and Chair Department of Global Health

Professor of Medicine

Emory University School of Medicine

Rollins School of Public Health

Atlanta, Georgia

Roy Gulick, MD, MPH

Rochelle Belfer Professor of Medicine

Chief, Division of Infectious Diseases

Weill Cornell Medicine

New York, New York

Donna M. Jacobsen, BS

Executive Director/President

IAS–USA

Executive Manager

Conference on Retroviruses and Opportunistic Infections (CROI)

San Francisco, California

Jeanne M. Marrazzo, MD, MPH

Professor of Medicine

Division of Infectious Diseases

University of Alabama at Birmingham

Birmingham, Alabama

Douglas D. Richman, MD

Professor of Pathology and Medicine

University of California San Diego and
Veterans Affairs San Diego Healthcare System

La Jolla, California

Michael Saag, MD

Professor of Medicine

Jim Straley Chair in AIDS Research

Director, Center for AIDS Research

Associate Dean for Global Health, School of Medicine

University of Alabama at Birmingham

Birmingham, Alabama

Robert Schooley, MD

Professor of Medicine

Head, Division of Infectious Diseases

Vice Chair, Department of Medicine

Senior Director, International Initiatives

University of California San Diego

La Jolla, California

**eBox 2. IAS–USA Antiretroviral Drug Resistance Testing Recommendations Panel**

Huldrych F. Günthard, MD (Panel Chair)

Professor of Infectious Diseases

President of the Swiss HIV Cohort Study

Deputy Chief, Division of Infectious Diseases and Hospital Epidemiology

University Hospital Zurich

Zurich, Switzerland

Vincent Calvez, MD, PhD

Hopital Pitie-Salpetriere

Paris, France

Donna M. Jacobsen, BS*

Executive Director/President

IAS–USA

Executive Manager

Conference on Retroviruses and Opportunistic Infections (CROI)

San Francisco, California

Roger Paredes, MD, PhD

Infectious Diseases Service and IrsiCaixa AIDS Research Institute

Hospital Universitari Germans Trias i Pujol

Badalona, Spain

Deenan Pillay, MD, PhD

Professor of Virology

University College London

Director of the Africa Centre for Health and Population Studies

University of KwaZulu Natal

Mtubatuba, South Africa

Douglas D. Richman, MD*

Professor of Pathology and Medicine

University of California San Diego and
Veterans Affairs San Diego Healthcare System

La Jolla, California

Robert W. Shafer, MD

Professor (Research) of Medicine (Infectious Diseases) and, by Courtesy, of Pathology

Stanford University Medical School

Stanford, California

Annemarie M. Wensing, MD, PhD

University Medical Center Utrecht

Utrecht, The Netherlands

*IAS–USA Board of Directors liaison

**eBox 3. Working Sections of the IAS–USA Antiretroviral Drug Resistance Testing Recommendations Panel**

Introduction

Huldrych Günthard, MD, and Douglas D. Richman, MD

Transmission, Origin, and Epidemiology of Drug Resistance

Huldrych Günthard, MD

Selection of Resistance With Low-Level Viremia

Annemarie Wensing, MD, PhD

Methods for Resistance Testing

Roger Paredes, MD, PhD

Effects of Subtype on HIV-1 Drug Resistance

Robert Shafer, MD

Drug Resistance Test Interpretation

Robert Shafer, MD, and Vincent Calvez, MD

Clinical Applications and Recommendations

Roger Paredes, MD, PhD

Use of HIV Drug Resistance Genetic Sequences for Other Purposes

Deenan Pillay, MD, PhD

Future Directions

Huldrych Günthard, MD, and Douglas D. Richman, MD

**eTable 1. Summary of Evidence Collection**

| **Evidence Identification** | **Number of References From the Initial Search** | **Number of References Considered Possibly Relevant (Ultimately)** |  |
| --- | --- | --- | --- |
| **2017 Submission** | | |  |
| Relevant published reports and meeting abstracts   - Pubmed and Embase searches  (03/2008 to 09/2017) | **>54,000** | **333** |  |
| - Panel members’ identification* | ongoing | |  |
| Number of relevant references reported in manuscript (initially submitted 198,) | | | **117** |

*Of note, individual panel members collected relevant evidence throughout the process, which cannot be quantified**.**

**eTable 2. Search Terms Used and Results of Embase and PubMed Literature Searches**

| Search | Search Term | Results |
| --- | --- | --- |
| #1 | **HIV Low Frequency** | **373** |
| #2 | **HIV minority variants** | **212** |
| #3 | **HIV next-generation sequencing** | **457** |
| #4 | **Allele-specific PCR** | **4128** |
| #5 | **HIV bioinformatics** | **2520** |
| #6 | **HIV databases** | **3226** |
| #7 | **HIV phylogenetics** | **86** |
| #8 | **(((HIV-1) AND Low-Level Viremia) AND Resistance in HIV-1 drug] AND HIV Low Frequency** | **9** |
| #9 | **HIV AND Drug Resistance AND (Test or Testing) AND (Interpretation OR Algorithm)** | **260** |
| #10 | **((((HIV-1_ AND drug resistance) AND subtype [Title/Abstract]) AND (“2008”[Date-Publication]: “3000” [Date- Publication])** | **914** |
| #11 | **HIV Transmission** | **49,985** |

**eTable 3.** **Strength of Recommendation and Quality of Evidence Rating Scale^a^**

| **Rating** | **Definition** |
| --- | --- |
| **Strength of recommendation** |  |
| A | Strong support for the recommendation |
| B | Moderate support for the recommendation |
| C | Limited support for the recommendation |
| **Quality of evidence** |  |
| Ia | Evidence from 1 or more randomized clinical trials  published in the peer-reviewed literature |
| Ib | Evidence from 1 or more randomized clinical trials  presented in abstract form at peer-reviewed scientific meetings |
| IIa | Evidence from nonrandomized clinical trials or cohort or case-control  studies published in the peer-reviewed literature |
| IIb | Evidence from nonrandomized clinical trials or cohort or case-control  studies presented in abstract form at peer-reviewed scientific meetings |
| III | Recommendation based on the panel’s analysis of the accumulated available evidence |

^a^Adapted in part from the Canadian Task Force on Periodic Health Examination, *Can Med Assoc J,* 1979.^21^

**Extended Discussion of Specific Topics According to Main Manuscript**

**ORIGIN OF MINORITY VARIANTS HARBORING DRM IN DRUG NAÏVE PATIENTS**

The presence of minority variants harboring DRM in drug naïve individuals may originate from one of 3 sources: incomplete reversion of TDR majority variants; transmission as minority variant harboring DRMs; spontaneously occurring DRM that can be identified by highly sensitive assays such as allele-specific PCR or next-generation sequencing (NGS). In chronically infected drug-naive individuals, a large proportion of such minority variants harboring DRMs represent the remaining fraction of the TDR strain after reversion to wildtype amino acid has occurred. The fitness advantage of wildtype virus in absence of drugs permits this virus to replace drug-resistant variants.^22,23^ Minority variants appearing during primary infection originate from transmission or spontaneous mutations.^24,25^

**EMERGENCE OF RESISTANCE WITH LOW-LEVEL VIREMIA**

Suppression of plasma viral load below the limits of quantification of routinely available viral load assays is a well-established objective of successful ART.^26^ Detectable viremia during ART between the limit of the assay quantification (20 or 50 copies/mL) and 1000 copies per mL is generally referred to as low-level viremia. If low level viremia is transient and is only observed in a single measurement followed by an undetectable viral load result in a subsequent measurement, it is termed a viral “blip”. Detectable viremia below the limit of quantification is often referred to as residual viremia or very low level viremia. The source and clinical relevance of both this very low level viremia and viral blips have not been clearly elucidated. Some of these measurements may be attributable to technical variability. In a large international comparison of more than 4000 paired viral load assay results, relatively good concordance was observed for higher quantification levels, but concordance around the lower limit of quantification is less consistent^27^ partially attributable to the greater coefficient of variation with lower measurements. Low level viremia and blips may also reflect real biological processes. One proposed mechanism is the release of virus from activated latently infected cells that in the presence of ART do not result in active rounds of virus replication. An alternative mechanism is a low level of ongoing virus replication due to poor adherence or to insufficient drug penetration in certain tissues and anatomical compartments.^28-33^ Low level replication is the only mechanism by which new drug resistance variants may be selected within the context of low level viremia.

Patients with very low level viremia during ART have in comparison to patients with more complete virus suppression on ART more often presented with a higher baseline viral load, a larger amount of proviral HIV DNA, a lower CD4 cell count, and more advanced disease. Very low level viremia has been reported to be a risk factor for viral rebound above 50 copies/mL, but this is not consistently observed in all studies and the number of reported patients with subsequent virologic failure or selection of HIV drug resistance are scarce. As such there is insufficient evidence to perform drug resistance testing on specimens with VLLV (below 50 copies/mL) in clinical practice (evidence rating AIIa).

Four recent large cohort studies and one clinical trial defining viral rebound as 2 viral load measurements of above 50 copies/mL or a single measurement above 1000 copies/mL did find a correlation between the magnitude of blips and subsequent failure, an observation that has not been observed in many smaller studies.^34-38^ Of note the majority of patients in these studies were treated with NNRTI- or PI-based ART, not with InSTIs. Emergence of resistance was not reported in these studies, but considering the lack of correlation with viral rebound there is no evidence to support performing drug resistance testing on samples with a single blip with an amplitude below 200 copies/mL in clinical practice (evidence rating BIIa). Samples with a blip exceeding 200 copies/mL could be considered for resistance testing if available (evidence rating CIII). Adherence counseling should be performed for all patients with viral blips (evidence rating AIII).

In several large cohorts, a significantly increased risk for virologic rebound was observed individuals with repeated measurements of detectable, low-level viremia during ART. In the large US-European Antiretroviral Therapy Cohort Collaboration (ART-CC) cohort^39^ (n= 17,902) an increased hazard ratio (HR) of 3.97 (95% CI, 3.05–5.17) of a confirmed rebound above 500 copies/mL or switch of therapy after single rebound was observed for low level viremia of 200 to 499 copies/mL, but did not reach statistical significance (HR, 1.38; 95% CI, 0.96-2.00) for the lowest range of 50 to 199 copies/mL. In a French cohort^40^ (n=2,374) low level viremia between 50 and 199 copies/mL conferred an increased hazard of a viral rebound above 200 copies/mL (HR, 2.30 95% CI, 1·65–3·20). In a British-German cohort (n= 1386)^41^ unstratified low level viremia between 50 and 400 copies/mL was associated with a viral rebound above 400 copies/mL. In resource limited settings the differentiation between blips and low level viremia is hampered by the low frequency of viral monitoring. One very large South African multi-center cohort study (n=70,930) reported that single detection of a viral load result of 51 to 999 copies/mL increases the hazard of a viral rebound above 1000 copies for all ranges of LLV (HR, 2.6; 95% CI 2.5-2.8), with risk most pronounced for the highest range of viremia (400-999 copies/L) (HR, 4.7; 95% CI, 4.2-5.2).^42^ Resuppression on the same regimen was frequently observed as well, indicating the importance of adherence counseling. One large single center long-term cohort study from Canada (n=1,357) observed similar hazard ratios for a rebound above 1000 copies/mL following persistent LLV (HR ranging from 2.22 to 4.83.^43^ None of these cohorts reported on selection of drug resistance during low level viremia.

Several studies have shown that drug resistance can develop in patients with levels of viremia below 1000 copies/mL. These studies utilized different thresholds for low level viremia and different criteria for resistance; however, each of these confirmed that drug resistance can emerge at diminished levels of HIV replication already in the range of 50 to 200 copies/mL with increased risk at higher levels in the presence of the selective pressure of ART.^44-46^

Although resistance assay kits are only approved by the FDA for viral loads above 1000 copies/mL, several studies have shown the feasibility of resistance testing at lower ranges of viremia.^26,44,46,47^ To avoid biases during the amplification recommendation process, more plasma can be used for the extraction of HIV RNA.

**EFFECT of SUBTYPE ON HIV-1 DRUG RESISTANCE**

HIV-1 group M viruses have evolved into numerous subtypes and circulating recombinant forms differing from each other by approximately 12% in HIV-1 *pol*. Although subtype B viruses account for just about 10% of the HIV-1 pandemic, as the predominant subtype in North America and Europe they have been disproportionately studied. As ART has expanded globally, the effect of subtype on HIV-1 drug resistance has received increasing attention.

Considering the relatively high conservation of HIV-1 *pol*, it is not surprising that all HIV-1 subtypes are highly susceptible to each of the approved nRTIs, NNRTIs, PIs, and InSTIs.^48^ For about 15 years, it has been a requirement for FDA approval to demonstrate that a new ARV is highly active against viruses belonging to all HIV-1 subtypes. Indeed, all published ARV treatment guidelines, including those of the US Public Health Service, IAS-USA, EACS and the WHO, provide recommendations that are similar for each subtype.

In contrast to the ARVs targeting virus enzymes, certain entry inhibitors may not be equally active against all virus subtypes because HIV-1 *env* is more variable than HIV-1 *pol.* For example, the investigational attachment inhibitor class is not uniformly active against all subtypes.^49^ Nonetheless, the fusion inhibitor enfuvirtide appears to be active against the vast majority of viruses belonging to different HIV-1 subtypes and the CCR5 inhibitor maraviroc is active against CCR5-tropic viruses regardless of subtype.^50,51^

Virtually all amino acid differences between subtypes are polymorphisms, variants that occur commonly in the absence of therapy.^52^ Although several of these polymorphisms may reduce ARV susceptibility in the presence of an established DRM, most appear to have little phenotypic or clinical effect when they occur alone. For example, the NNRTI-associated mutation E138A which reduces rilpivirine susceptibility by about 2-fold, and the InSTI-associated mutation T97A which reduces elvitegravir susceptibility by about 5-fold each occur in about 1% to 5% of viruses depending on subtype.^53,54^ However, the clinical significance of these mutations in previously untreated individuals is uncertain.^53,54^ Minor PI mutations, mostly polymorphisms, were not associated with treatment failure in ART-naive, subtype-B infected patients treated with an initial PI-containing regimen^55^).

With several exceptions, most HIV-1 DRMs occur in similar proportions in different subtypes.^56^ Perhaps the most important exception is the increased propensity of subtype C viruses to develop the nRTI-resistance mutation K65R. Subtype C viruses have a polyadenine stretch between codons 63 and 65, which predisposes the virus to substitute a guanosine triphosphate (GTP) for an adenosine trisphosphate (ATP) at the second position of codon 65 after tenofovir exposure in vitro^57,58^ and in vivo.^59-61^

Most other examples of preferentially selected DRMs result from inter-subtype differences in codon usage. For example, the NNRTI-resistance mutation V106M occurs more often in subtype C viruses than in other subtypes, because V106M requires a single base-pair change in subtype C viruses, GTG (V) => ATG (M), but a double base-pair change in all other subtypes, GTA (V) => ATG (M).^62^ The propensity to develop this otherwise uncommon NNRTI-resistance mutation is relevant because point-mutation assays for subtype C viruses have had to include probes for detecting V106M.^63,64^

The wild-type glycine at integrase position 140 of subtype B viruses is more likely to be encoded by GGC or GGT (rather than GGA or GGG). As a result, subtype B viruses require a single GTP to ATP change at the first position of the G140 codon to develop the accessory InSTI-resistance mutation G140S. G140S is an important compensatory mutation for the DRMs Q148H/R/K, which may explain why Q148 mutations appear to occur more commonly in individuals with subtype B viruses receiving raltegravir than the alternative InSTI-resistance pathway characterized by N155H, which is more common in non-B viruses.^65^ This propensity may prove to be clinically significant because the combination of G140S plus Q148H/R/K is the foundation for high-level dolutegravir resistance.

Virus subtype may have subtle effects on how DRMs influence HIV-1 susceptibility.^66-68^ Although no DRM has been reported to reproducibly produce a discernable subtype-specific difference in drug susceptibility, this possibility cannot be excluded because most published phenotypic data are based on phenotypic assays in which patient-derived *pol* genes are transferred into a subtype B virus vector.^69^ Despite evidence that HIV-1 *gag* contains determinants of PI resistance,^70,71^ it remains uncertain whether subtype-specific recombinant virus assays will improve the reliability of susceptibility testing.^66^

Individual ARVs and standard ART regimens appear to be similarly active in treating ART-naive individuals regardless of subtype.^55,59,72-77^ Nonetheless, several studies have described an increased risk of virologic failure associated with a particular subtype.^78-81^

Such studies are often confounded by geographic location, treatment facilities, and clinical, social and economic status that could affect these outcome differences among individuals infected with different subtypes. A causal basis for a subtype-specific risk of treatment failure would be more compelling if a consistent virologic or resistance mechanism that explained the risk could be demonstrated.

HIV-2 infects more than 1 million persons, most of whom reside in or have emigrated from West Africa. It differs from HIV-1 by more than 50% of its genome. HIV-2 is intrinsically resistant to NNRTIs and is variably susceptible to the PIs.^82,83^ HIV-1 group O is a non-pandemic HIV-1 variant that is also found primarily in West Africa. A high proportion of group O viruses are also resistant to NNRTIs. Although group O viruses appear susceptible to the other 5 ART drug classes, their level of susceptibility to current ART drugs may differ from group M viruses.^84,85^

**METHODS FOR HIV-1 RESISTANCE TESTING**

**Resistance Test Options**

Sanger-based and next generation sequencing (NGS) approaches are both suited for genotypic testing. The choice of method depends on availability, laboratory expertise, equipment and bioinformatics support. Of note, technology is constantly evolving (See **eBox 4**).

The proportion of the virus population with DRMs in an individual that will compromise the efficacy of a particular drug is not well defined; nevertheless, some conclusions can be made: 1) The presence of more than 1% to 2% DRMs reflects a robust technical threshold for NGS.^86^ 2) The detection of 15% to 20% of DRMs by Sanger sequencing corresponds with high concordance to NGS. 3) There is a continuous dose-effect relationship between the amount of low-frequency mutants, as determined by multiplying the HIV viral load by the proportion of drug resistant variants, and the risk of virologic failure to initial NNRTI-based ART regimen.^23^ A nationwide study in Mexico suggested that a 5% mutant threshold provides the best discrimination between individuals who do or do not respond to initial NNRTI-containing ART.^87^ A similar threshold has not been defined for other drug classes or for ART-experienced individuals, for whom the potential benefit of NGS over Sanger sequencing remains undefined.^88,89^

Until clinically meaningful cutoff values are confirmed, a conservative approach is recommended that for NGS-derived data to consider a specimen resistant if 15% to 20% of viruses in the sample have a DRM as can be discerned with Sanger sequencing (evidence rating AIIa). However, drug resistance data obtained using a lower sensitivity threshold (ie, 1%-5% [evidence rating BIIa]) may help decide whether NNRTIs might be used.^23,90-94^ In any case, genotyping centers should store all mutation information down to the 1% NGS technical threshold to enable future characterization and validation of clinically relevant sensitivity cutoffs for different drugs and treatment scenarios.

The recommended compartment for drug resistance testing is plasma. The bulk of evidence associating viral resistance with clinical outcomes has been generated with plasma sequencing. In individuals with low-level viremia (ie, <200 copies/mL) peripheral blood mononuclear cell (PBMC) sequencing is technically feasible and more likely to provide an HIV genotype than plasma. However, the PBMC compartment contains HIV DNA that has been archived throughout the patient’s infection, and may be highly discordant with plasma testing. Clinicians should judge results from PBMC testing with caution.

**Genotypic Resistance Test Interpretation**

Genotypic resistance interpretation systems can be divided into rule-based and machine-learning systems. Rule-based systems require a knowledge base and a set of derived rules. The knowledge base usually comprises studies of the relationship between a DRM and an antiretroviral drug including (i) whether the drug selects the DRM, (ii) whether the DRM reduces drug activity in vitro, and (iii) whether the DRM reduces the virologic response to an ART regimen containing the drug.

Studies that report whether a drug selects a DRM in vitro or in patients often provide the first evidence that the DRM reduces drug susceptibility. Site-directed mutagenesis studies are used to confirm whether a DRM specifically reduces drug susceptibility by comparing the in vitro susceptibility results of isogenic viruses with and without a DRM. In addition, correlating genotypes with phenotypes can characterize the impact of a DRM on cross-resistance to different antiretroviral drugs in the same class.

Virologic response studies have been most often performed in the context of clinical trials.^95^ Such studies, for example, have assessed the effect of nRTI-associated DRMs on the virologic response to regimens containing abacavir or tenofovir disoproxil fumarate (TDF);^96,97^ PI-associated DRMs on the response to regimens containing lopinavir/ritonavir and darunavir/ritonavir;^98,99^ NNRTI-associated DRMs on the response to regimens containing etravirine;^100^ and InSTI-associated DRMs on the response to regimens containing dolutegravir.^101^

Rule-based systems have been used more commonly than machine-learning systems for HIV-1 resistance interpretation because of their ability to consider diverse forms of data and to incorporate expert opinion.^102-105^ These systems have the advantage of being reproducible, transparent, and educational, but have the disadvantage of being subjective. Well-described rule-based systems include the French National Agency for Research on AIDS and Viral Hepatitis (ANRS) system, the Rega system, HIV GRADE, and the Stanford HIV Drug Resistance Database system.^105-107^ Although these systems may produce somewhat different estimates of drug resistance for the same drug, their overall predictive ability generally has been similar.^103,104,108^ An online system for interpreting HIV-2 sequences has also been developed.^109^

Machine-learning systems have used datasets containing large amounts of data, for example, correlating the DRMs in a sequence with reduced susceptibility^110-112^ or with the virologic response to a new treatment regimen.^113,114^ Given a dataset with a sufficiently large number of such correlations, these systems can use genotypic data to predict fold-reductions in susceptibility^111,112^ or the likelihood of virologic suppression with a new regimen.^110,113,114^

**Entry Inhibitor Resistance**

One mechanism of resistance to the CCR5 coreceptor (R5) inhibitor maraviroc arises when the suppression of R5-tropic viruses results in the outgrowth of chemokine receptor CXCR4 (X4)-tropic variants present at low levels before therapy with maraviroc.^115^ These preexisting X4-tropic variants can be detected using phenotypic or genotypic assays capable of detecting minority X4-tropic variants.) Genotypic determination of HIV-1 coreceptor usage is based on sequencing of the V3-coding region of HIV-1 *env*, the principal, but not only, determinant of coreceptor usage.^116^ A second mechanism of maraviroc resistance is the emergence of mutations that enable R5-tropic variants to adapt to a maraviroc-bound R5 coreceptor.^117^ This mechanism results from different unpredictable combinations of HIV-1 *env* changes that make it detectable only phenotypically.^117^ Resistance to the fusion inhibitor enfuvirtide can be detected genotypically by sequencing the region of gp41 encompassing amino acid residues 36 to 45, which contain the main enfuvirtide-associated DRMs.

**Novel formulations.** The 2 tenofovir prodrugs, TDF and tenofovir alafenamide (TAF), select for the same DRMs. Because TAF results in higher levels of the active anabolite, tenofovir diphosphate, than does TDF,^118^ it may eventually be shown to have a higher barrier to resistance than TDF. In addition, TAF may confer more partial activity than TDF as tenofovir-associated DRMs accumulate. Today, resistance interpretations for TAF and TDF remain the same.

**Proviral DNA sequencing.** Because proviral DNA remains detectable in PBMCs of patients with an undetectable plasma HIV-1 RNA level after ART, PBMC genotyping is often considered in those with complete virologic suppression who require a change in therapy.^6^ There is a strong but imperfect correlation between the DRMs in PBMC proviral DNA and a patient’s past treatment history and previous genotypic test results.^119-121^ However, PBMC sequencing may not necessarily detect all the DRMs previously present in samples from patients who had past genotypic tests.^119,121^ Therefore, PBMC genotypic resistance testing should be used in conjunction with past genotypic test results, with results interpreted in light of drug resistance likely to have emerged and archived during past episodes of virologic failure.

**USE OF GENETIC SEQUENCES FOR OTHER PURPOSES**

**Transmission Networks**

Phylogenetic methods allow for identification of transmission “clusters” of infections, which for example help to assess the relative spread of virus within and between risk groups.^122-126^ The application of such approaches in real time can also inform targeted public health prevention measures for high risk transmitters, as described for injection drug users,^127^ and modelled for MSM*.*^128^ Other approaches have sought to quantify the contribution of those at different stages of infection (eg, undiagnosed, acute infection, and therapy failure) to ongoing transmissions.^14,129,130^ Many public health agencies now routinely utilize these approaches as a component of HIV surveillance efforts. A key focus in resource-limited settings is the role of mobile populations in maintaining the epidemic, as has been described in Uganda*.*^131^

**Epidemic Growth Characteristics**

Through phylodynamics, in which consideration of the date virus is sampled is added to phylogenetic linkage approaches,^132^ it is possible to map the origin and rate of growth of specific epidemics. This method has been applied to date the origin and routes of spread of HIV-1.^133,134^ It can also be used to map the spread of specific genotypes, termed phylotypes, for instance, to demonstrate the long-lasting persistence and spread of drug-resistant viruses among treatment-naive individuals,^17,18,134,135^ an important contribution to overall rates of transmitted drug resistance.

**Incidence Measures**

HIV infection is characterized by transmission of a single or small number of viral clones. The quasispecies then diversifies within that individual over time. Because HIV gene sequencing can identify minority viral species, there is some evidence that this viral diversity, as measured by sequencing, can estimate the time since infection.^136^ NGS has the capacity to correctly characterize the quasispecies with greater depth, and, therefore, enhance the ability to measure diversity.^137^ It may also increase the power to identify the direction of transmission between 2 individuals, something currently impossible to glean strictly from consensus sequences generated by Sanger sequencing.

Although phylogenetic methods provide major opportunities to support public health measures to prevent transmission of HIV, the potential to uncover transmission networks comes with a risk as HIV stigma remains widespread. In some countries, knowingly transmitting HIV is a criminal offense. In others, sex between men is illegal. For these reasons, the storage of HIV genetic sequences and application of phylogenetics should be undertaken within a rigorous ethics framework. Infected individuals who contribute their samples for analysis must receive assurance that they will not be adversely affected by the use of such data.

**eBox 4. Sanger and Next-Generation Sequencing (NGS) Methods**

| **Sanger sequencing** of variants present in an individual with HIV-infection at a given time has been the most frequently used and clinically validated genotypic resistance test available. Sanger sequencing-based genotypes are simple to perform and analyze by a laboratory technician using semi-automated drug resistance interpretation methods. Several clinical laboratory improvement amendments (CLIA)-certified commercial assays provide technically and clinically validated wet lab kits. Genotyping costs can be greatly reduced using home-brew methods,^138^ and may be applicable in low- and middle-income countries will find appealing. The primary limitation of Sanger sequencing is that it only detects mutants present in 15% to 20% of the virus population and can miss potentially relevant information for constructing effective ART combinations.^23,92,139^  **NGS** platforms are rapidly evolving toward increased robustness, decreased complexity, and reduced cost.^91,93^ These platforms all perform parallel sequencing of as many as millions of individual DNA molecules. The number of sequence reads obtained reflects the testing of the polymerase chain reaction (PCR)-amplified product generated from the clinical specimen. The viral load in the specimen and the amount of specimen analyzed will determine the number of molecules of HIV-1 RNA that actually get tested. The numerous sequence reads also permits quantification of clonal viral variants from the same sample with higher sensitivity than Sanger sequencing and at a lower cost per base.^140-143^ NGS can be more affordable than Sanger sequencing when sequencing a sufficiently large number of samples in a single run.^144^ Moreover, NGS provides increased sensitivity to detect low-abundance drug-resistant mutations and allows testing more regions of the HIV-1 genome at minimal additional cost. Academic centers and private companies are developing semi-automated bioinformatics analysis pipelines that will allow NGS analyses to be performed by non-expert laboratory technicians.^145^ Resistance testing by PCR and subsequent PCR amplification of HIV-1 RNA molecules cannot be avoided with current NGS platforms; PCR-derived error is the major factor limiting NGS sensitivity regardless of the NGS platform used. PCR-derived recombination precludes reliable linkage of different mutations on single genomes with routine clinical testing.  **Point mutation assays (PMA)**, including allele-specific PCR,^86,146-148^ oligonucleotide ligation assay,^149^ or LigAmp,^150^ among others, are a promising approach to future point-of-care drug resistance assays,^63^ but currently have no utility for routine HIV genotyping. |
| --- |

References

1. Carpenter CCJ, Fischl MA, Hammer SM, et al. Antiretroviral therapy for HIV infection in 1996: recommendations of an international panel. *JAMA.* 1996;276(2):146-154.

2. Carpenter CCJ, Fischl MA, Hammer SM, et al. Antiretroviral therapy for HIV infection in 1997: updated recommendations of the International AIDS Society–USA panel. *JAMA.* 1997;277:1962-1969.

3. Marrazzo JM, del Rio C, Holtgrave DR, et al. HIV prevention in clinical care settings: 2014 recommendations of the International Antiviral Society-USA panel. *JAMA.* 2014;312(4):390-409.

4. Günthard HF, Saag MS, Benson CA, et al. Antiretroviral drugs for treatment and prevention of HIV infection in adults: 2016 recommendations of the International Antiviral Society-USA panel. *JAMA.* 2016;316(2):191-210.

5. Martin DF, Dunn JP, Davis JL, et al. Use of the ganciclovir implant for the treatment of cytomegalovirus retinitis in the era of potent antiretroviral therapy: recommendations of the International AIDS Society–USA panel. *Am J Ophthalmol.* 1999;127(3):329-339.

6. Saag MS, Holodniy M, Kuritzkes DR, et al. HIV viral load markers in clinical practice. *Nat Med.* 1996;2(6):625-629.

7. Whitley RJ, Jacobson MA, Friedberg DN, et al. Guidelines for the treatment of cytomegalovirus diseases in patients with AIDS in the era of potent antiretroviral therapy. *Arch Intern Med.* 1998;158:957-969.

8. Schambelan M, Benson CA, Carr A, et al. Management of metabolic complications associated with antiretroviral therapy for HIV-1 infection: recommendations of an International AIDS Society-USA panel. *JAIDS.* 2002;31(3):257-275.

9. Hirsch MS, Günthard HF, Schapiro JM, et al. Antiretroviral drug resistance testing in adult HIV-1 infection: 2008 recommendations of an International AIDS Society-USA panel. *Clin Infect Dis.* 2008;47(2):266-285.

10. Hirsch MS, Brun-Vézinet F, Clotet B, et al. Antiretroviral drug resistance testing in adults infected with human immunodeficiency virus type I: 2003 recommendations of an International AIDS Society-USA panel. *Clin Infect Dis.* 2003;37:113-128.

11. Hirsch MS, Brun-Vézinet F, D'Aquila RT, et al. Antiretroviral drug resistance testing in adult HIV-1 infection: recommendations of an International AIDS Society-USA Panel. *JAMA.* 2000;283(18):2417-2426.

12. Hirsch MS, Conway B, D'Aquila RT, et al. Antiretroviral drug resistance testing in adults with HIV infection: implications for clinical management. International AIDS Society–USA Panel. *JAMA.* 1998;279(24):1984-1991.

13. Yeni PG, Hammer SM, Hirsch MS, et al. Treatment for adult HIV infection: 2004 recommendations of the International AIDS Society-USA panel. *JAMA.* 2004;292:251-265.

14. Yeni PG, Hammer SM, Carpenter CCJ, et al. Antiretroviral treatment for adult HIV-1 infection in 2002: updated recommendations of the International AIDS Society-USA panel. *JAMA.* 2002;288:222-235.

15. Thompson MA, Aberg JA, Hoy JF, et al. Antiretroviral treatment of adult HIV infection: 2012 recommendations of the International Antiviral Society-USA panel. *JAMA.* 2012;308(4):387-402.

16. Thompson MA, Aberg JA, Cahn P, et al. Antiretroviral treatment of adult HIV infection: 2010 recommendations of the International AIDS Society-USA panel. *JAMA.* 2010;304(3):321-333.

17. Hammer SM, Saag MS, Schechter M, et al. Treatment for adult HIV infection: 2006 recommendations of the International AIDS Society-USA panel. *JAMA.* 2006;296:827-843.

18. Hammer SM, Eron JJ, Jr., Reiss P, et al. Antiretroviral treatment of adult HIV infection: 2008 recommendations of the International AIDS Society-USA panel. *JAMA.* 2008;300(5):555-570.

19. Carpenter CCJ, Cooper DA, Fischl MA, et al. Antiretroviral therapy for HIV infection in adults: updated recommendations of the International AIDS Society–USA panel. *JAMA.* 2000;283(3):381-390.

20. Carpenter CCJ, Fischl MA, Hammer SM, et al. Antiretroviral therapy for HIV infection in 1998: updated recommendations of the International AIDS Society–USA panel. *JAMA.* 1998;280:78-86.

21. Canadian Task Force on the Periodic Health Examination. The periodic health examination. *Can Med Assoc J.* 1979;121(9):1193-1254.

22. Bansal V, Metzner KJ, Niederost B, et al. Minority K65R variants and early failure of antiretroviral therapy in HIV-1-infected Eritrean immigrant. *Emerg Infect Dis.* 2011;17(10):1966-1968.

23. Li JZ, Paredes R, Ribaudo HJ, et al. Low-frequency HIV-1 drug resistance mutations and risk of NNRTI-based antiretroviral treatment failure: a systematic review and pooled analysis. *JAMA.* 2011;305(13):1327-1335.

24. Metzner KJ, Scherrer AU, Preiswerk B, et al. Origin of minority drug-resistant HIV-1 variants in primary HIV-1 infection. *J Infect Dis.* 2013;208(7):1102-1112.

25. Gianella S, Delport W, Pacold ME, et al. Detection of minority resistance during early HIV-1 infection: natural variation and spurious detection rather than transmission and evolution of multiple viral variants. *J Virol.* 2011;85(16):8359-8367.

26. Ryscavage P, Kelly S, Li J, et al. Significance and clinical management of persistent low-level viremia and very-low-level viremia in HIV-1-infected patients. *Antimicrob Agents Chemother.* 2015;58(7):3585-3598.

27. Swenson LC, Cobb B, Geretti AM, et al. Comparative performances of HIV-1 RNA load assays at low viral load levels: results of an international collaboration. *J Clin Microbiol.* 2014;52(2):517-523.

28. Gonzalez-Serna A, Swenson LC, Watson B, et al. A single untimed plasma drug concentration measurement during low-level HIV viremia predicts virologic failure. *Clin Microbiol Infect.* 2016;22(12):1004.

29. Leierer G, Grabmeier-Pfistershammer K, Steuer A, et al. A Single Quantifiable Viral Load Is Predictive of Virological Failure in Human Immunodeficiency Virus (HIV)-Infected Patients on Combination Antiretroviral Therapy: The Austrian HIV Cohort Study. *Open Forum Infect Dis.* 2016;3(2):ofw089.

30. Maggiolo F, Di FE, Comi L, et al. Reduced adherence to antiretroviral therapy is associated with residual low-level viremia. *Pragmat Obs Res.* 2017;8:91-97.

31. Trezza CR, Kashuba AD. Pharmacokinetics of antiretrovirals in genital secretions and anatomic sites of HIV transmission: implications for HIV prevention. *Clin Pharmacokinet.* 2014;53(7):611-624.

32. Baheti G, Kiser JJ, Havens PL, Fletcher CV. Plasma and intracellular population pharmacokinetic analysis of tenofovir in HIV-1-infected patients. *Antimicrob Agents Chemother.* 2011;55(11):5294-5299.

33. Wynn HE, Brundage RC, Fletcher CV. Clinical implications of CNS penetration of antiretroviral drugs. *CNS Drugs.* 2002;16(9):595-609.

34. Young J, Rickenbach M, Calmy A, et al. Transient detectable viremia and the risk of viral rebound in patients from the Swiss HIV Cohort Study. *BMC Infect Dis.* 2015;15:382.

35. Porter DP, Kulkarni R, Garner W, Miller MD, White KL. Viral blips were infrequent in treatment-naive adults treated with rilpivirine/emtricitabine/tenofovir DF or efavirenz/emtricitabine/tenofovir DF through 96 weeks. *Antivir Ther.* 2017;

36. Erdbeer G, Sabranski M, Sonntag I, et al. Intermittent viraemia and immune reconstitution in patients with more than 10-15 years of antiretroviral therapy: baseline values still matter. *J Int AIDS Soc.* 2014;17(4 Suppl 3):19689.

37. Farmer A, Wang X, Ganesan A, et al. Factors associated with HIV viral load "blips" and the relationship between self-reported adherence and efavirenz blood levels on blip occurrence: a case-control study. *AIDS Res Ther.* 2016;13:16.

38. Sorstedt E, Nilsson S, Blaxhult A, et al. Viral blips during suppressive antiretroviral treatment are associated with high baseline HIV-1 RNA levels. *BMC Infect Dis.* 2016;16:305.

39. Vandenhende MA, Ingle S, May M, et al. Impact of low-level viremia on clinical and virological outcomes in treated HIV-1-infected patients. *AIDS.* 2015;29(3):373-383.

40. Vandenhende MA, Perrier A, Bonnet F, et al. Risk of virological failure in HIV-1-infected patients experiencing low-level viraemia under active antiretroviral therapy (ANRS C03 cohort study). *Antivir Ther.* 2015;20(6):655-660.

41. Geretti AM, Smith C, Haberl A, et al. Determinants of virological failure after successful viral load suppression in first-line highly active antiretroviral therapy. *Antivir Ther.* 2008;13(7):927-936.

42. Hermans P, et al. Effect of HIV-1 low-level viremia during antiretroviral therapy on treatment outcomes in WHO-guided South African treatment programmes: a multicentre cohort study. *Lancet Infect Dis*. In press.

43. Laprise C, de PA, Baril JG, Dufresne S, Trottier H. Virologic failure following persistent low-level viremia in a cohort of HIV-positive patients: results from 12 years of observation. *Clin Infect Dis.* 2013;57(10):1489-1496.

44. Gonzalez-Serna A, Min JE, Woods C, et al. Performance of HIV-1 Drug Resistance Testing at Low-Level Viremia and Its Ability to Predict Future Virologic Outcomes and Viral Evolution in Treatment-Naive Individuals. *Clin Infect Dis.* 2014;58(8):1165-1173.

45. Tang MW, Liu TF, Shafer RW. The HIVdb system for HIV-1 genotypic resistance interpretation. *Intervirology.* 2012;55(2):98-101.

46. Swenson LC, Min JE, Woods CK, et al. HIV drug resistance detected during low-level viraemia is associated with subsequent virologic failure. *AIDS.* 2014;28(1125):1134.

47. Taiwo B, Gallien S, Aga E, et al. Antiretroviral drug resistance in HIV-1-infected patients experiencing persistent low-level viremia during first-line therapy. *J Infect Dis.* 2011;204(4):515-520.

48. Parkin NT, Schapiro JM. Antiretroviral drug resistance in non-subtype B HIV-1, HIV-2 and SIV. *Antivir Ther.* 2004;9(1):3-12.

49. Zhou N, Nowicka-Sans B, McAuliffe B, et al. Genotypic correlates of susceptibility to HIV-1 attachment inhibitor BMS-626529, the active agent of the prodrug BMS-663068. *J Antimicrob Chemother.* 2014;69(3):573-581.

50. Araujo LA, Almeida SE. HIV-1 diversity in the envelope glycoproteins: implications for viral entry inhibition. *Viruses.* 2013;5(2):595-604.

51. Panos G, Watson DC. Effect of HIV-1 subtype and tropism on treatment with chemokine coreceptor entry inhibitors; overview of viral entry inhibition. *Crit Rev Microbiol.* 2015;41(4):473-487.

52. Rhee SY, Sankaran K, Varghese V, et al. HIV-1 Protease, Reverse Transcriptase, and Integrase Variation. *J Virol.* 2016;90(13):6058-6070.

53. Abram ME, Ram RR, Margot NA, et al. Lack of impact of pre-existing T97A HIV-1 integrase mutation on integrase strand transfer inhibitor resistance and treatment outcome. *PLoS One.* 2017;12(2):e0172206.

54. Sluis-Cremer N, Jordan MR, Huber K, et al. E138A in HIV-1 reverse transcriptase is more common in subtype C than B: implications for rilpivirine use in resource-limited settings. *Antiviral Res.* 2014;107:31-34.

55. Scherrer AU, Ledergerber B, von W, V, et al. Improved virological outcome in White patients infected with HIV-1 non-B subtypes compared to subtype B. *Clin Infect Dis.* 2011;53(11):1143-1152.

56. Rhee SY, Varghese V, Holmes SP, et al. Mutational Correlates of Virological Failure in Individuals Receiving a WHO-Recommended Tenofovir-Containing First-Line Regimen: An International Collaboration. *EBioMedicine.* 2017;18:225-235.

57. Invernizzi CF, Coutsinos D, Oliveira M, Moisi D, Brenner BG, Wainberg MA. Signature nucleotide polymorphisms at positions 64 and 65 in reverse transcriptase favor the selection of the K65R resistance mutation in HIV-1 subtype C. *J Infect Dis.* 2009;200(8):1202-1206.

58. Coutsinos D, Invernizzi CF, Xu H, et al. Template usage is responsible for the preferential acquisition of the K65R reverse transcriptase mutation in subtype C variants of human immunodeficiency virus type 1. *J Virol.* 2009;83(4):2029-2033.

59. White E, Smit E, Churchill D, et al. No Evidence That HIV-1 Subtype C Infection Compromises the Efficacy of Tenofovir-Containing Regimens: Cohort Study in the United Kingdom. *J Infect Dis.* 2016;214(9):1302-1308.

60. TenoRes Study Group. Global epidemiology of drug resistance after failure of WHO recommended first-line regimens for adult HIV-1 infection: a multicentre retrospective cohort study. *Lancet Infect Dis.* 2016;16(5):565-575.

61. Theys K, Vercauteren J, Snoeck J, et al. HIV-1 subtype is an independent predictor of reverse transcriptase mutation K65R in HIV-1 patients treated with combination antiretroviral therapy including tenofovir. *Antimicrob Agents Chemother.* 2013;57(2):1053-1056.

62. Brenner B, Turner D, Oliveira M, et al. A V106M mutation in HIV-1 clade C viruses exposed to efavirenz confers cross-resistance to non-nucleoside reverse transcriptase inhibitors. *AIDS.* 2003;17:F1-F5.

63. Rhee SY, Jordan MR, Raizes E, et al. HIV-1 Drug Resistance Mutations: Potential Applications for Point-of-Care Genotypic Resistance Testing. *PLoS One.* 2015;10(12):e0145772.

64. Chung MH, Beck IA, Dross S, et al. Oligonucleotide ligation assay detects HIV drug resistance associated with virologic failure among antiretroviral-naive adults in Kenya. *JAIDS.* 2014;67(3):246-253.

65. Doyle T, Dunn DT, Ceccherini-Silberstein F, et al. Integrase inhibitor (INI) genotypic resistance in treatment-naive and raltegravir-experienced patients infected with diverse HIV-1 clades. *J Antimicrob Chemother.* 2015;70(11):3080-3086.

66. Bronze M, Steegen K, Wallis CL, et al. HIV-1 phenotypic reverse transcriptase inhibitor drug resistance test interpretation is not dependent on the subtype of the virus backbone. *PLoS One.* 2012;7(4):e34708.

67. Derache A, Wallis CL, Vardhanabhuti S, Bartlett J, Kumarasamy N, Katzenstein D. Phenotype, Genotype, and Drug Resistance in Subtype C HIV-1 Infection. *J Infect Dis.* 2016;213(2):250-256.

68. Basson AE, Rhee SY, Parry CM, et al. Impact of drug resistance-associated amino acid changes in HIV-1 subtype C on susceptibility to newer nonnucleoside reverse transcriptase inhibitors. *Antimicrob Agents Chemother.* 2015;59(2):960-971.

69. Petropoulos CJ, Parkin NT, Limoli KL, et al. A novel phenotypic drug susceptibility assay for human immunodeficiency virus type 1. *Antimicrob Agents Chemother.* 2000;44:920-928.

70. Fun A, Wensing AM, Verheyen J, Nijhuis M. Human Immunodeficiency Virus Gag and protease: partners in resistance. *Retrovirology.* 2012;9:63.

71. Sutherland KA, Mbisa JL, Cane PA, Pillay D, Parry CM. Contribution of Gag and protease to variation in susceptibility to protease inhibitors between different strains of subtype B human immunodeficiency virus type 1. *J Gen Virol.* 2014;95(Pt 1):190-200.

72. Rockstroh JK, Teppler H, Zhao J, et al. Clinical efficacy of raltegravir against B and non-B subtype HIV-1 in phase III clinical studies. *AIDS.* 2011;25(11):1365-1369.

73. Geretti AM, Harrison L, Green H, et al. Effect of HIV-1 subtype on virologic and immunologic response to starting highly active antiretroviral therapy. *Clin Infect Dis.* 2009;48(9):1296-1305.

74. Dierynck I, De MS, Lathouwers E, et al. In vitro susceptibility and virological outcome to darunavir and lopinavir are independent of HIV type-1 subtype in treatment-naive patients. *Antivir Ther.* 2010;15(8):1161-1169.

75. Antiretroviral Therapy Cohort Collaboration (ART-CC), Canadian Observational Cohort Collaboration (CANOC), UK Collaboration HIV Cohort Study (UK CHIC), Collaboration of Observational HIV Epidemiological Research in Europe (COHERE). Mortality of treated HIV-1 positive individuals according to viral subtype in Europe and Canada: collaborative cohort analysis. *AIDS.* 2016;30(3):503-513.

76. Chaix ML, Seng R, Frange P, et al. Increasing HIV-1 non-B subtype primary infections in patients in France and effect of HIV subtypes on virological and immunological responses to combined antiretroviral therapy. *Clin Infect Dis.* 2013;56(6):880-887.

77. Bannister WP, Ruiz L, Loveday C, et al. HIV-1 subtypes and response to combination antiretroviral therapy in Europe. *Antivir Ther.* 2006;11:707-715.

78. Kyeyune F, Nankya I, Metha S, et al. Treatment failure and drug resistance is more frequent in HIV-1 subtype D versus subtype A-infected Ugandans over a 10-year study period. *AIDS.* 2013;27(12):1899-1909.

79. Easterbrook PJ, Smith M, Mullen J, et al. Impact of HIV-1 viral subtype on disease progression and response to antiretroviral therapy. *J Int AIDS Soc.* 2010;13:4.

80. Haggblom A, Svedhem V, Singh K, Sonnerborg A, Neogi U. Virological failure in patients with HIV-1 subtype C receiving antiretroviral therapy: an analysis of a prospective national cohort in Sweden. *Lancet HIV.* 2016;3(4):e166-e174.

81. Kantor R, Smeaton L, Vardhanabhuti S, et al. Pretreatment HIV Drug Resistance and HIV-1 Subtype C Are Independently Associated With Virologic Failure: Results From the Multinational PEARLS (ACTG A5175) Clinical Trial. *Clin Infect Dis.* 2015;60(10):1541-1549.

82. Ekouevi DK, Tchounga BK, Coffie PA, et al. Antiretroviral therapy response among HIV-2 infected patients: a systematic review. *BMC Infect Dis.* 2014;14:461.

83. Menendez-Arias L, Alvarez M. Antiretroviral therapy and drug resistance in human immunodeficiency virus type 2 infection. *Antiviral Res.* 2014;102:70-86.

84. Tebit DM, Patel H, Ratcliff A, et al. HIV-1 Group O Genotypes and Phenotypes: Relationship to Fitness and Susceptibility to Antiretroviral Drugs. *AIDS Res Hum Retroviruses.* 2016;32(7):676-688.

85. Mourez T, Simon F, Plantier JC. Non-M variants of human immunodeficiency virus type 1. *Clin Microbiol Rev.* 2013;26(3):448-461.

86. Paredes R, Marconi VC, Campbell TB, Kuritzkes DR. Systematic evaluation of allele-specific real-time PCR for the detection of minor HIV-1 variants with pol and env resistance mutations. *J Virol Methods.* 2007;146(1-2):136-146.

87. Avila-Rios S, Garcia-Morales C, Matias-Florentino M, et al. Pretreatment HIV-drug resistance in Mexico and its impact on the effectiveness of first-line antiretroviral therapy: a nationally representative 2015 WHO survey. *Lancet HIV.* 2016;3(12):e579-e591.

88. Charpentier C, Lee GQ, Rodriguez C, et al. Highly frequent HIV-1 minority resistant variants at baseline of the ANRS 139 TRIO trial had a limited impact on virological response. *J Antimicrob Chemother.* 2015;70(7):2090-2096.

89. Pou C, Noguera-Julian M, Perez-Alvarez S, et al. Improved prediction of salvage antiretroviral therapy outcomes using ultrasensitive HIV-1 drug resistance testing. *Clin Infect Dis.* 2014;59(4):578-588.

90. Gianella S, Richman DD. Minority variants of drug-resistant HIV. *J Infect Dis.* 2010;202(5):657-666.

91. Casadella M, Paredes R. Deep sequencing for HIV-1 clinical management. *Virus Res.* 2016;

92. Paredes R, Lalama CM, Ribaudo HJ, et al. Pre-existing minority drug-resistant HIV-1 variants, adherence, and risk of antiretroviral treatment failure. *J Infect Dis.* 2010;201(5):662-671.

93. Geretti AM, Paredes R, Kozal MJ. Transmission of HIV drug resistance: lessons from sensitive screening assays. *Curr Opin Infect Dis.* 2015;28(1):23-30.

94. Cozzi-Lepri A, Noguera-Julian M, Di GF, et al. Low-frequency drug-resistant HIV-1 and risk of virological failure to first-line NNRTI-based ART: a multicohort European case-control study using centralized ultrasensitive 454 pyrosequencing. *J Antimicrob Chemother.* 2015;70(3):930-940.

95. Brun-Vezinet F, Costagliola D, Khaled MA, et al. Clinically validated genotype analysis: guiding principles and statistical concerns. *Antivir Ther.* 2004;9(4):465-478.

96. Miller MD, Margot N, Lu B, et al. Genotypic and phenotypic predictors of the magnitude of response to tenofovir disoproxil fumarate treatment in antiretroviral-experienced patients. *J Infect Dis.* 2004;189(5):837-846.

97. Lanier ER, Ait-Khaled M, Scott J, et al. Antiviral efficacy of abacavir in antiretroviral therapy-experienced adults harbouring HIV-1 with specific patterns of resistance to nucleoside reverse transcriptase inhibitors. *Antivir Ther.* 2004;9(1):37-45.

98. King MS, Rode R, Cohen-Codar I, et al. Predictive genotypic algorithm for virologic response to lopinavir-ritonavir in protease inhibitor-experienced patients. *Antimicrob Agents Chemother.* 2007;51:3067-3074.

99. De Meyer S, Vangeneugden T, van BB, et al. Resistance profile of darunavir: combined 24-week results from the POWER trials. *AIDS Res Hum Retroviruses.* 2008;24(3):379-388.

100. Vingerhoets J, Tambuyzer L, Azijn H, et al. Resistance profile of etravirine: combined analysis of baseline genotypic and phenotypic data from the randomized, controlled Phase III clinical studies. *AIDS.* 2010;24(4):503-514.

101. Eron JJ, Clotet B, Durant J, et al. Safety and efficacy of dolutegravir in treatment-experienced subjects with raltegravir-resistant HIV type 1 infection: 24-week results of the VIKING Study. *J Infect Dis.* 2013;207(5):740-748.

102. Vercauteren J, Beheydt G, Prosperi M, et al. Clinical evaluation of Rega 8: an updated genotypic interpretation system that significantly predicts HIV-therapy response. *PLoS One.* 2013;8(4):e61436.

103. Frentz D, Boucher CA, Assel M, et al. Comparison of HIV-1 genotypic resistance test interpretation systems in predicting virological outcomes over time. *PLoS One.* 2010;5(7):e11505.

104. Rhee SY, Fessel WJ, Liu TF, et al. Predictive value of HIV-1 genotypic resistance test interpretation algorithms. *J Infect Dis.* 2009;200(3):453-463.

105. Paredes R, Tzou PL, van ZG, et al. Collaborative update of a rule-based expert system for HIV-1 genotypic resistance test interpretation. *PLoS One.* 2017;12(7):e0181357.

106. Obermeier M, Pironti A, Berg T, et al. HIV-GRADE: a publicly available, rules-based drug resistance interpretation algorithm integrating bioinformatic knowledge. *Intervirology.* 2012;55(2):102-107.

107. Eberle J, Gurtler L. The evolution of drug resistance interpretation algorithms: ANRS, REGA and extension of resistance analysis to HIV-1 group O and HIV-2. *Intervirology.* 2012;55(2):128-133.

108. Fox ZV, Geretti AM, Kjaer J, et al. The ability of four genotypic interpretation systems to predict virological response to ritonavir-boosted protease inhibitors. *AIDS.* 2007;21:2033-2042.

109. Charpentier C, Camacho R, Ruelle J, et al. HIV-2EU: supporting standardized HIV-2 drug resistance interpretation in Europe. *Clin Infect Dis.* 2013;56(11):1654-1658.

110. Beerenwinkel N, Montazeri H, Schuhmacher H, et al. The individualized genetic barrier predicts treatment response in a large cohort of HIV-1 infected patients. *PLoS Comput Biol.* 2013;9(8):e1003203.

111. Altmann A, Daumer M, Beerenwinkel N, et al. Predicting the response to combination antiretroviral therapy: retrospective validation of geno2pheno-THEO on a large clinical database. *J Infect Dis.* 2009;199(7):999-1006.

112. Vermeiren H, Van CE, Alen P, Bacheler L, Picchio G, Lecocq P. Prediction of HIV-1 drug susceptibility phenotype from the viral genotype using linear regression modeling. *J Virol Methods.* 2007;145(1):47-55.

113. Revell AD, Wang D, Boyd MA, et al. The development of an expert system to predict virological response to HIV therapy as part of an online treatment support tool. *AIDS.* 2011;25(15):1855-1863.

114. Winters B, Montaner J, Harrigan PR, et al. Determination of clinically relevant cutoffs for HIV-1 phenotypic resistance estimates through a combined analysis of clinical trial and cohort data. *J Acquir Immune Defic Syndr.* 2008;48(1):26-34.

115. Archer J, Rambaut A, Taillon BE, Harrigan PR, Lewis M, Robertson DL. The evolutionary analysis of emerging low frequency HIV-1 CXCR4 using variants through time--an ultra-deep approach. *PLoS Comput Biol.* 2010;6(12):e1001022.

116. Vandekerckhove LP, Wensing AM, Kaiser R, et al. European guidelines on the clinical management of HIV-1 tropism testing. *Lancet Infect Dis.* 2011;11(5):394-407.

117. Jiang X, Feyertag F, Meehan CJ, et al. Characterizing the Diverse Mutational Pathways Associated with R5-Tropic Maraviroc Resistance: HIV-1 That Uses the Drug-Bound CCR5 Coreceptor. *J Virol.* 2015;89(22):11457-11472.

118. Margot NA, Liu Y, Miller MD, Callebaut C. High resistance barrier to tenofovir alafenamide is driven by higher loading of tenofovir diphosphate into target cells compared to tenofovir disoproxil fumarate. *Antiviral Res.* 2016;132:50-58.

119. Delaugerre C, Braun J, Charreau I, et al. Comparison of resistance mutation patterns in historical plasma HIV RNA genotypes with those in current proviral HIV DNA genotypes among extensively treated patients with suppressed replication. *HIV Med.* 2012;13(9):517-525.

120. Diallo K, Murillo WE, de Rivera IL, et al. Comparison of HIV-1 resistance profiles in plasma RNA versus PBMC DNA in heavily treated patients in Honduras, a resource-limited country. *Int J Mol Epidemiol Genet.* 2012;3(1):56-65.

121. Wirden M, Soulie C, Valantin MA, et al. Historical HIV-RNA resistance test results are more informative than proviral DNA genotyping in cases of suppressed or residual viraemia. *J Antimicrob Chemother.* 2011;66(4):709-712.

122. Turk T, Bachmann N, Kadelka C, et al. Assessing the danger of self-sustained HIV epidemics in heterosexuals by population based phylogenetic cluster analysis. *Elife.* 2017;6

123. von W, V, Kouyos RD, Yerly S, et al. The role of migration and domestic transmission in the spread of HIV-1 non-B subtypes in Switzerland. *J Infect Dis.* 2011;204(7):1095-1103.

124. Kouyos RD, von W, V, Yerly S, et al. Molecular epidemiology reveals long-term changes in HIV type 1 subtype B transmission in Switzerland. *J Infect Dis.* 2010;201(10):1488-1497.

125. Wertheim JO, Leigh Brown AJ, Hepler NL, et al. The global transmission network of HIV-1. *J Infect Dis.* 2014;209(2):304-313.

126. Lewis F, Hughes GJ, Rambaut A, Pozniak A, Leigh Brown AJ. Episodic sexual transmission of HIV revealed by molecular phylodynamics. *PLoS Med.* 2008;5(3):e50.

127. Poon AF, Gustafson R, Daly P, et al. Near real-time monitoring of HIV transmission hotspots from routine HIV genotyping: an implementation case study. *Lancet HIV.* 2016;3(5):e231-e238.

128. Little SJ, Kosakovsky Pond SL, Anderson CM, et al. Using HIV networks to inform real time prevention interventions. *PLoS One.* 2014;9(6):e98443.

129. Ratmann O, van SA, Bezemer D, et al. Sources of HIV infection among men having sex with men and implications for prevention. *Sci Transl Med.* 2016;8(320):320ra2.

130. Fisher M, Pao D, Brown AE, et al. Determinants of HIV-1 transmission in men who have sex with men: a combined clinical, epidemiological and phylogenetic approach. *AIDS.* 2010;24(11):1739-1747.

131. Grabowski MK, Lessler J, Redd AD, et al. The role of viral introductions in sustaining community-based HIV epidemics in rural Uganda: evidence from spatial clustering, phylogenetics, and egocentric transmission models. *PLoS Med.* 2014;11(3):e1001610.

132. Grenfell BT, Pybus OG, Gog JR, et al. Unifying the epidemiological and evolutionary dynamics of pathogens. *Science.* 2004;303(5656):327-332.

133. Gilbert MT, Rambaut A, Wlasiuk G, Spira TJ, Pitchenik AE, Worobey M. The emergence of HIV/AIDS in the Americas and beyond. *Proc Natl Acad Sci U S A.* 2007;104(47):18566-18570.

134. Faria NR, Rambaut A, Suchard MA, et al. HIV epidemiology. The early spread and epidemic ignition of HIV-1 in human populations. *Science.* 2014;346(6205):56-61.

135. Hue S, Gifford RJ, Dunn D, Fernhill E, Pillay D. Demonstration of sustained drug-resistant human immunodeficiency virus type 1 lineages circulating among treatment-naive individuals. *J Virol.* 2009;83(6):2645-2654.

136. Kouyos RD, von W, V, Yerly S, et al. Ambiguous nucleotide calls from population-based sequencing of HIV-1 are a marker for viral diversity and the age of infection. *Clin Infect Dis.* 2011;52(4):532-539.

137. Puller V, Neher R, Albert J. Estimating time of HIV-1 infection from next-generation sequence diversity. *PLoS Comput Biol.* 2017;13(10):e1005775.

138. Inzaule SC, Ondoa P, Peter T, et al. Affordable HIV drug-resistance testing for monitoring of antiretroviral therapy in sub-Saharan Africa. *Lancet Infect Dis.* 2016;16(11):e267-e275.

139. Metzner KJ, Rauch P, Walter H, et al. Detection of minor populations of drug-resistant HIV-1 in acute seroconverters. *AIDS.* 2005;19:1819-1825.

140. Metzker ML. Sequencing technologies - the next generation. *Nat Rev Genet.* 2010;11(1):31-46.

141. Goodwin S, McPherson JD, McCombie WR. Coming of age: ten years of next-generation sequencing technologies. *Nat Rev Genet.* 2016;17(6):333-351.

142. Eisenstein M. Startups use short-read data to expand long-read sequencing market. *Nat Biotechnol.* 2015;33(5):433-435.

143. Chabria SB, Gupta S, Kozal MJ. Deep sequencing of HIV: clinical and research applications. *Annu Rev Genomics Hum Genet.* 2014;15:295-325.

144. Lapointe HR, Dong W, Lee GQ, et al. HIV drug resistance testing by high-multiplex "wide" sequencing on the MiSeq instrument. *Antimicrob Agents Chemother.* 2015;59(11):6824-6833.

145. Noguera-Julian M, Edgil D, Harrigan PR, Sandstrom P, Godfrey C, Paredes R. Next-generation human immunodeficiency virus sequencing for patient management and drug resistance surveillance. *J Infect Dis.* 2017;

146. Halvas EK, Aldrovandi GM, Balfe P, et al. Blinded, multicenter comparison of methods to detect a drug-resistant mutant of human immunodeficiency virus type 1 at low frequency. *J Clin Microbiol.* 2006;44:2612-2614.

147. Hauser A, Mugenyi K, Kabasinguzi R, Kuecherer C, Harms G, Kunz A. Emergence and persistence of minor drug-resistant HIV-1 variants in Ugandan women after nevirapine single-dose prophylaxis. *PLoS One.* 2011;6(5):e20357.

148. Metzner KJ, Rauch P, von W, V, et al. Efficient suppression of minority drug-resistant HIV type 1 (HIV-1) variants present at primary HIV-1 infection by ritonavir-boosted protease inhibitor-containing antiretroviral therapy. *J Infect Dis.* 2010;201(7):1063-1071.

149. Micek MA, Blanco AJ, Beck IA, et al. Nevirapine resistance by timing of HIV type 1 infection in infants treated with single-dose nevirapine. *Clin Infect Dis.* 2010;50(10):1405-1414.

150. Flys TS, Mwatha A, Guay LA, et al. Detection of K103N in Ugandan women after repeated exposure to single dose nevirapine. *AIDS.* 2007;21(15):2077-2082.
